# Decreased SIRT1 expression in the peripheral blood of patients with Graves’ disease

**DOI:** 10.1530/JOE-19-0501

**Published:** 2020-06-02

**Authors:** Qinglei Yin, Liyun Shen, Yicheng Qi, Dalong Song, Lei Ye, Ying Peng, Yanqiu Wang, Zhou Jin, Guang Ning, Weiqing Wang, Dongping Lin, Shu Wang

**Affiliations:** 1 Shanghai National Clinical Research Center for Endocrine and Metabolic Diseases, Key Laboratory for Endocrine and Metabolic Diseases of the National Health Commission of the PR China, Shanghai Institute of EndocrineRuijin Hospital, Shanghai Jiao-Tong University School of Medicine, China; 2 Division of Endocrinology and Metabolism, Department of Internal Medicine, RenJi Hospital, Shanghai Jiao-Tong University School of Medicine, Pudong, Shanghai, China; 3 Reproductive Medicine Center, Guangdong Provincial People’s Hospital, Guangdong Academy of Medical Science, Guangzhou, China; 4 Department of Endocrinology and Metabolism, Shanghai Ninth People’s Hospital, Affiliated Shanghai Jiao-Tong University School of Medicine, Shanghai, China

**Keywords:** thyroid, Graves’ disease, SIRT1, NF-κB

## Abstract

SIRT1, a class III histone/protein deacetylase (HDAC), has been associated with autoimmune diseases. There is a paucity of data about the role of SIRT1 in Graves’ disease. The aim of this study was to investigate the role of SIRT1 in the pathogenesis of GD. Here, we showed that SIRT1 expression and activity were significantly decreased in GD patients compared with healthy controls. The NF-κB pathway was activated in the peripheral blood of GD patients. The reduced SIRT1 levels correlated strongly with clinical parameters. In euthyroid patients, SIRT1 expression was markedly upregulated and NF-κB downstream target gene expression was significantly reduced. SIRT1 inhibited the NF-κB pathway activity by deacetylating P65. These results demonstrate that reduced SIRT1 expression and activity contribute to the activation of the NF-κB pathway and may be involved in the pathogenesis of GD.

## Introduction

Graves’ disease (GD) is the most common cause of hyperthyroidism, in which patients develop an anti-thyroid autoimmune response, including lymphocytic infiltration and the presence of autoantibodies against thyroglobulin, thyroid peroxidase and the thyroid-stimulating hormone receptor (TSHR) (Weetman 2000). In addition to genetic and environmental factors, immune malfunctions may also be involved in the development of GD. It has been suggested that the interaction of antigen-presenting cells, thyroid follicular cells and autoreactive T cells causes an autoimmune response against thyroid antigens (Weetman 2004). Although agitation of the adaptive immune system and the destruction of self-tolerance has been observed, the fundamental mechanism remains unclear.

SIRT1 is a class III histone/protein deacetylase (HDAC) and a member of the silent information regulator (Sir2) family. The mammalian sirtuin SIRT1 has received much attention for the roles it plays in regulating metabolism and protecting against age-related diseases (Haigis & Sinclair 2010). At the cellular level, SIRT1 regulates a variety of processes, including autophagy (Salminen & Kaarniranta 2009), energy homeostasis (Rodgers *et al.* 2005), mitochondrial biogenesis (Scarpulla 2011), and apoptosis (Luo *et al.* 2001, Son *et al.* 2019).

Recently, a report identified polymorphisms in the *SIRT1* gene associated with the increased production of thyroid autoantibodies (Sarumaru *et al.* 2016). Furthermore, SIRT1 has emerged as a critical immune modulator that acts by suppressing inflammation or regulating immune cell activation. In antigen-presenting cells, SIRT1 inhibits the production of proinflammatory cytokines (Yang *et al.* 2015). SIRT1 directs the differentiation of myeloid-derived suppressor cells (MDSCs) in antitumor immunity (Liu *et al.* 2014*a*). SIRT1 may also negatively regulate T-cell activation via the deacetylation of the promoter region to inhibit the transcription of Bclaf1 (Zhang *et al.* 2009, Gao *et al.* 2012, Kong *et al.* 2011). Moreover, SIRT1 is a critical suppressor of T-cell immunity that acts by suppressing the activity of transcription factors, such as nuclear factor kappa light chain enhancer of activated B cells (NF-κB) and AP-1 (Fei *et al.* 2015).

NF-κΒ is initially located in the cytoplasm in an inactive form complexed with inhibitor of kappa B (IκB), an NF-κΒ inhibitor. Following cellular stimulation, IκB proteins become phosphorylated by IκB kinase (IKK), which subsequently targets IκB for ubiquitination and degradation through the 26S proteasome (Ghosh *et al.* 2002). Consequently, NF-κB is released from the complex and translocates to the nucleus, where it interacts with specific DNA-recognition sites to mediate gene transcription (Lawrence 2009). NF-κΒ signaling is modulated by posttranslational modifications (PTMs) (Perkins 2006). PTMs are integral components of gene expression programs. To date, >200 different PTMs have been identified that influence diverse aspects of signaling regulation (Hirsch *et al.* 2016). PTMs also act as critical regulators of cellular signal transduction during immune responses (Deribe *et al.* 2010, Liu *et al.* 2016). In addition to conventional PTMs, such as phosphorylation and ubiquitination, which have been extensively elucidated in cellular signaling pathways, other unconventional PTMs, such as acetylation and methylation, are increasingly being shown to control immune and inflammatory responses (Mowen & David 2014, Cao 2016, Li *et al.* 2016, Chen *et al.* 2017). Protein acetylation has a variety of effects, including regulating enzymatic activity, protein-protein interactions, nucleic acid binding, protein stability, and subcellular localization (Gu & Roeder 1997, Ageta-Ishihara *et al.* 2013, Wang *et al.* 2016). There are five main acetylation sites identified within P65. The acetylation of Lys^310^ is required for the full transcriptional activity of P65 (Yeung *et al.* 2004). Consequently, NF-κB-dependent transactivation depends on the balance between the acetylation and deacetylation status of NF-κB. SIRT1-mediated deacetylation can inhibit P65 function, and the deletion of SIRT1 can increase P65 acetylation and activity (Yeung *et al.* 2004).

The deacetylation of P65 by SIRT1 plays an important role in the development of disease. Previous studies have shown that SIRT1 is involved in diabetic kidney disease (Liu *et al.* 2014*b*) and rheumatoid arthritis (Li *et al.* 2018). In addition, the SIRT1 activator resveratrol has been shown to prevent and treat spontaneous type 1 diabetes, which normally develops in non-obese diabetic (NOD) mice (Lee *et al.* 2011). In contrast, there is a paucity of studies regarding the role of SIRT1 in GD pathogenesis.

Here, we investigated SIRT1 expression, activation and inhibition in peripheral blood mononuclear cells (PBMCs) extracted from GD patients and healthy controls. We found that SIRT1 deficiency plays a critical role in the activation of NF-κB during the pathogenesis of GD. The blockade of SIRT1 exacerbates the inflammatory response, whereas the activation of SIRT1 restores the expression of NF-κΒ target genes. The findings of the present study might provide new therapeutic target for the treatment of GD.

## Materials and methods

### Study subjects

Fresh blood was obtained from 51 patients with GD, 17 patients with Hashimoto’s thyroiditis (HT) and 30 age- and sex-matched healthy controls. GD was diagnosed based on clinical symptoms, biochemical indicators of hyperthyroidism and anti-thyroid-stimulating hormone receptor antibody (TRAb) positivity. HT was diagnosed based on anti-thyroid peroxidase antibody positivity, anti-thyroglobulin antibody positivity and ultrasound changes. All patients were recruited from Department of Endocrinology, Ruijin Hospital affiliated to Shanghai Jiao Tong University School of Medicine. Subjects with any chronic disease, infectious disease, cancer, diabetes or a family history of diabetes were excluded from this study. Besides, healthy controls had no family history of thyroid autoimmune disease or other autoimmune diseases. Clinical parameters, including thyrotropin (TSH), free T3 (FT3), free T4 (FT4), thyroperoxidase antibody (TPOAb), thyroglobulin antibody (TgAb) and thyrotropin receptor antibody (TRAb) levels, were obtained by routine clinical laboratory methods. The subject characteristics and clinical information of GD are shown in Table 1. The subject characteristics and clinical information of HT are shown in Supplementary Table 1, see section on supplementary materials given at the end of this article. A follow-up analysis of 15 GD patients with anti-thyroid therapy was performed. Patients on methimazole (MMI) therapy received 20–30 mg/day for the first phase, and the dose was reduced to 5–15 mg when remission was achieved in the patients. All the patients received more than 3 months of therapy. When the patients were euthyroid, their peripheral blood samples were obtained. The main clinical data of these follow-up patients are shown in Supplementary Table 2. All participants gave written informed consent in accordance with the Declaration of Helsinki. The study was approved by the Research Ethics Board of Ruijin Hospital.

**Table 1:** The clinical characteristics of patients with Graves’ disease and healthy controls.

Variable	HC	GD	Normal range
*n*	30	51	–
Age (years)	35 ± 12	36 ± 14	–
Gender (M/F)	8/22	12/39	–
FT3 (pmol/L)	4.2 ± 0.5	30.6 ± 14.0	2.63–5.70
FT4 (pmol/L)	12.6 ± 2.8	42.7 ± 12.3	9.01–19.04
TSH (μIU/mL)	2.19 ± 0.85	0.0012 ± 0.002	0.3500–4.9400
TRAb (IU/L)	–	15.1 ± 8.8	1.75
TPOAb (IU/mL)	1.30 ± 1.71	336.65 ± 356.21	<5.61
TGAb (IU/mL)	0.79 ± 1.19	194.67±307.15	<4.11

Data are expressed as mean ± s.d. according to the distribution.

F, female; GD, Graves’ disease; HC, healthy control; M, male.

‘–’ represents that the experiment was not performed or that the data are not available.

### Cell isolation

Human PBMCs were obtained from freshly collected blood in heparinized tubes and isolated by Ficoll-Isopaque density gradient centrifugation (Sigma-Aldrich). After centrifugation, the pellet was washed free of platelets and Ficoll. The isolated cells were used for further research.

### RNA isolation and RT-PCR

Total RNA was extracted by TRIzol reagent (Invitrogen) according to the manufacturer’s protocols. The cDNA was synthesized from 1 μg of RNA by reverse transcriptase (TaKaRa) with oligo dT-adaptor primers. Duplicate samples for quantitative PCR were run in an ICycler (ABI). The quantification of the expression of a given gene, expressed as the relative mRNA level compared with that of the control, was calculated with the 2^−ΔΔCt^ comparative method after normalization to the housekeeping gene GAPDH. Primer sequences are shown in Supplementary Table 3.

### Immunohistochemistry

Thyroid tissues were subjected to immunohistochemical analysis. Thyroid tissues were obtained from three patients with GD who were undergoing a thyroidectomy for treatment. Thyroid tissues from three patients with simple goiter were used as the control. Immunohistochemistry was performed as previously described (Hasegawa *et al.* 2010). Briefly, thyroid tissues were fixed in 4% paraformaldehyde overnight at 4°C, embedded in paraffin and cut into 5-μm thick sections. All sections were incubated with a rabbit anti-SIRT1 polyclonal antibody (Abcam, ab220807) at a 1:200 dilution overnight at 4°C. The sections were stained with biotin-labeled goat anti-rabbit IgG (Maixin Biotech, Fuzhou, China) and then with a streptavidin-peroxidase complex (Maixin Biotech). Next, 3,3′-diaminobenzidine (DAB; Maixin Biotech) was added to the samples. Finally, the sliced sections were counterstained, dehydrated, rinsed, and mounted in neutral gum.

### Immunofluorescence

PBMCs were spun onto glass slides using a cytocentrifuge 7620 (WESCOR, Logan, UT, USA) by centrifugation at 230 ***g*** for 5 min. Cytospins were stained with SIRT1 (Abcam, ab220807) or P65 (Cell Signaling Technology) antibodies. Fluorescent secondary antibodies were purchased from Invitrogen. Slides were mounted with Vectashield containing DAPI (SouthernBiotech, Birmingham, AL, USA). For fluorescence microscopy, all sections were stained and analyzed at the same time to exclude artifacts due to the variable decay of the fluorochrome. The images were acquired using an Olympus system.

### Western blotting analysis

Cell lysates were subjected to Western blotting analysis according to standard protocols. After being blocked, the membranes were incubated overnight at 4°C with primary antibodies against SIRT1 (Abcam, ab220807), acetyl-P65 (Abcam) and IκBα (Cell Signaling Technology). GAPDH (Cell Signaling Technology) was used as a normalization control. Next, the membranes were incubated with horseradish peroxidase-conjugated secondary antibodies (Cell Signaling Technology). Blots were developed with enhanced chemiluminescence substrate (Millipore), and detection was performed using LAS-4000 (GE Healthcare).

### SIRT1 deacetylase activity assay

The *in vitro* SIRT1 deacetylase activity was determined using a SIRT1 activity assay kit (Genmed, Shanghai, China) according to the manufacturer’s protocol. In brief, PBMCs were incubated with a synthesized substrate (Arg-His-Lys-Lys (Ac)). When SIRT1 is active, the acetylated substrate is deacetylated, resulting in a change in colorimetric absorbance. The absorbance was detected by a plate reader (Biotek).

### ELISA

Serum and cell supernatant levels of TNF-α, IL-6, IL-8 and MCP1 were measured using commercially available ELISA kits (R&D Systems). The procedures were performed in accordance with the manufacturer’s instructions. The absorbance was measured at a wavelength of 450 nm using a microplate reader to analyze the intensity of color development in each well.

### Cells and treatments

PBMCs were cultured in RPMI 1640 medium supplemented with 10% fetal bovine serum, 2 mM L-glutamine, 100 IU/mL penicillin and 100 μg/mL streptomycin at 37° in a humidified 5% CO_2_ atmosphere. All these components were purchased from Gibco. The cells were diluted with complete medium to a concentration of 1 × 10^6^/mL. The PBMCs were then seeded in 12-well plates, serum-starved for 12 h, and then treated with SIRT1 inhibitor Ex527 (40 μM; Selleckchem, Houston, TX, USA) and SRT1720, a putative SIRT1 activator (2 μM; Selleckchem). Lipopolysaccharide (LPS; *E. coli* 0111:B4, Sigma-Aldrich) was administered to cells at 50 ng/mL 5 h before cells were harvested.

P65-specific and scrambled siRNAs were designed and constructed by Genomeditech Co. Ltd. (Shanghai, China). The siRNAs were transfected into PBMCs according to the instructions provided with Lipofectamine 3000 (Invitrogen).

### Luciferase assays

HEK 293T cells were plated in 24-well plates 24 h before transfection. When the cells reached 40–50% confluence, they were transfected with a plasmid containing the promoter sequence of NF-κB using Lipofectamine 3000 transfection reagent (Invitrogen). Ex527 (40 μM; Selleckchem) and SRT1720 (2 μM; Selleckchem) were added at the same time. Twenty-four hours later, the cells were lysed in 1 × PLB and luciferase assays were performed using the Dual-Luciferase Reporter Assay System (Promega) as recommended by the manufacturer. All luciferase assay experiments were performed in triplicate.

### Data analysis

All statistical analyses were performed using SPSS Software 19.0 (SPSS Statistics Inc.). The normally distributed data were analyzed by independent-samples *t*-test. The analysis of abnormally distributed data was performed using the Kruskal–Wallis test followed by the Mann–Whitney *U*-test. Associations were analyzed using the Spearman correlation test. A paired *t*-test was used for comparing matched datasets. All graphs were generated using GraphPad Prism 7.0. A *P* value less than 0.05 was considered statistically significant.

## Results

### Reduced SIRT1 expression and activity in GD patients

**Figure 1:**
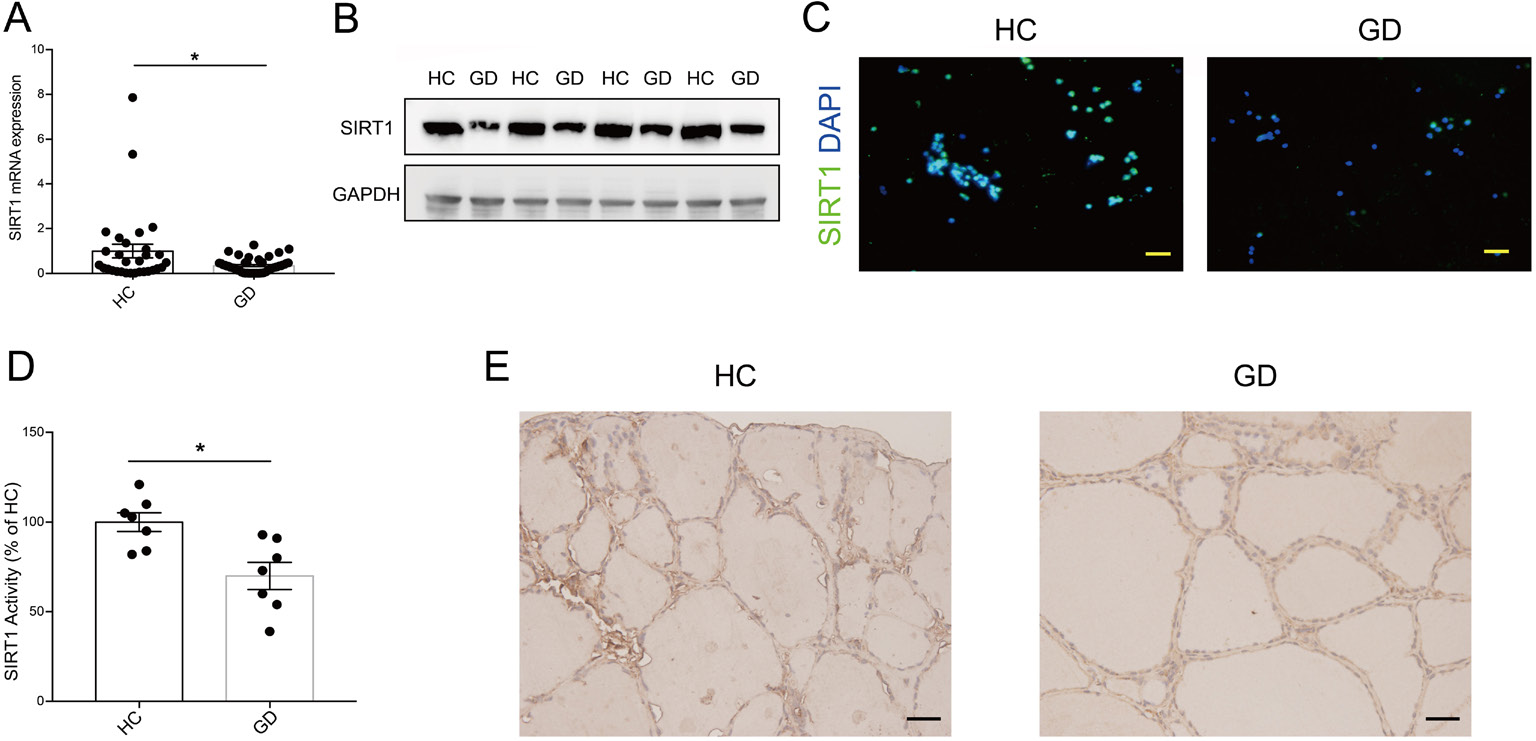
Reduced SIRT1 expression and activity in GD patients. (A) The levels of *SIRT1* mRNA in PBMCs were detected by qRT-PCR from 51 GD patients and 30 HCs. (B) Western blotting analysis of SIRT1 expression in GD patients and HC PBMCs (*n* = 4). (C) Representative images of immunofluorescence of SIRT1 (green) in GD patients and HC PBMCs. Nuclei were stained with DAPI (blue) (*n* = 5). Scale bars, 20 μm. (D) Total SIRT1 activity was determined in GD patients and HC PBMCs (*n* = 7). (E) Representative immunohistochemistry staining for SIRT1 in the thyroids of patients with GD and HCs (*n* = 3). Scale bars, 20 μm. GD, Graves’ disease; HC, healthy control. Data represent means ± s.e.m. *P < 0.05.

To investigate whether SIRT1 is involved in the pathogenesis of GD, we first measured whether SIRT1 was positive in CD4+ T, CD8+T, B-lymphocytes and monocytes which were the main composition of PBMCs, and we found SIRT1 was positive in them all (Supplementary Fig. 1). Then we examined the expression level of *SIRT1* mRNA in PBMCs obtained from 51 GD patients and 30 healthy controls (HCs) by qRT-PCR. The mRNA level of *SIRT1* was significantly decreased in GD patients compared with HCs (*P* < 0.05, Fig. 1A). Interestingly, the expression was also decreased in Hashimoto’s thyroiditis patients (Supplementary Table 1, Supplementary Fig. 2), indicating decreased SIRT1 is a common phenomenon in autoimmune thyroiditis (AITD). Furthermore, SIRT1 protein levels in PBMCs were reduced in GD patients compared to HCs (Fig. 1B), which was further confirmed by immunofluorescence in PBMCs (Fig. 1C). Next, we compared SIRT1 activity in PBMCs of GD and HC patients; SIRT1 activity was reduced in GD patient PBMCs (Fig. 1D). Immunohistochemical staining was used to measure SIRT1 protein expression levels in thyroid tissues collected from three GD patients and three HCs. Indeed, reduced SIRT1 expression was detected in the thyrocytes from patients with GD (Fig. 1E).

In patients with GD, TRAb is a typical autoantibody that binds to TSHR, thereby stimulating the synthesis and secretion of thyroid hormone and thyroid growth (Weetman 2000). As shown in Table 2, a negative correlation was found between *SIRT1* expression and TRAb in 51 GD patients (*r* = −0.512, *P* = 0.000, Table 2). Moreover, there was an inverse correlation between FT3 (*r* = −0.514, *P* = 0.000, Table 2) and FT4 (*r* = −0.394, *P* = 0.004, Table 2). These data clearly reveal that decreased SIRT1 expression is associated with GD clinical variables. Taken together, these data indicate that reduced SIRT1 expression might play a role in GD.

**Table 2:** The association between *SIRT1* mRNA expression and Graves’ disease clinical parameters by Spearman correlation.

GD parameters	*r*	*P*
FT3	−0.514	0.000
FT4	−0.394	0.004
TSH	0.211	0.137
TRAb	−0.512	0.000
TPOAb	0.040	0.779
TGAb	−0.047	0.745

*r*, correlation coefficient.

### NF-κΒ signaling pathway is activated in GD patients

**Figure 2:**
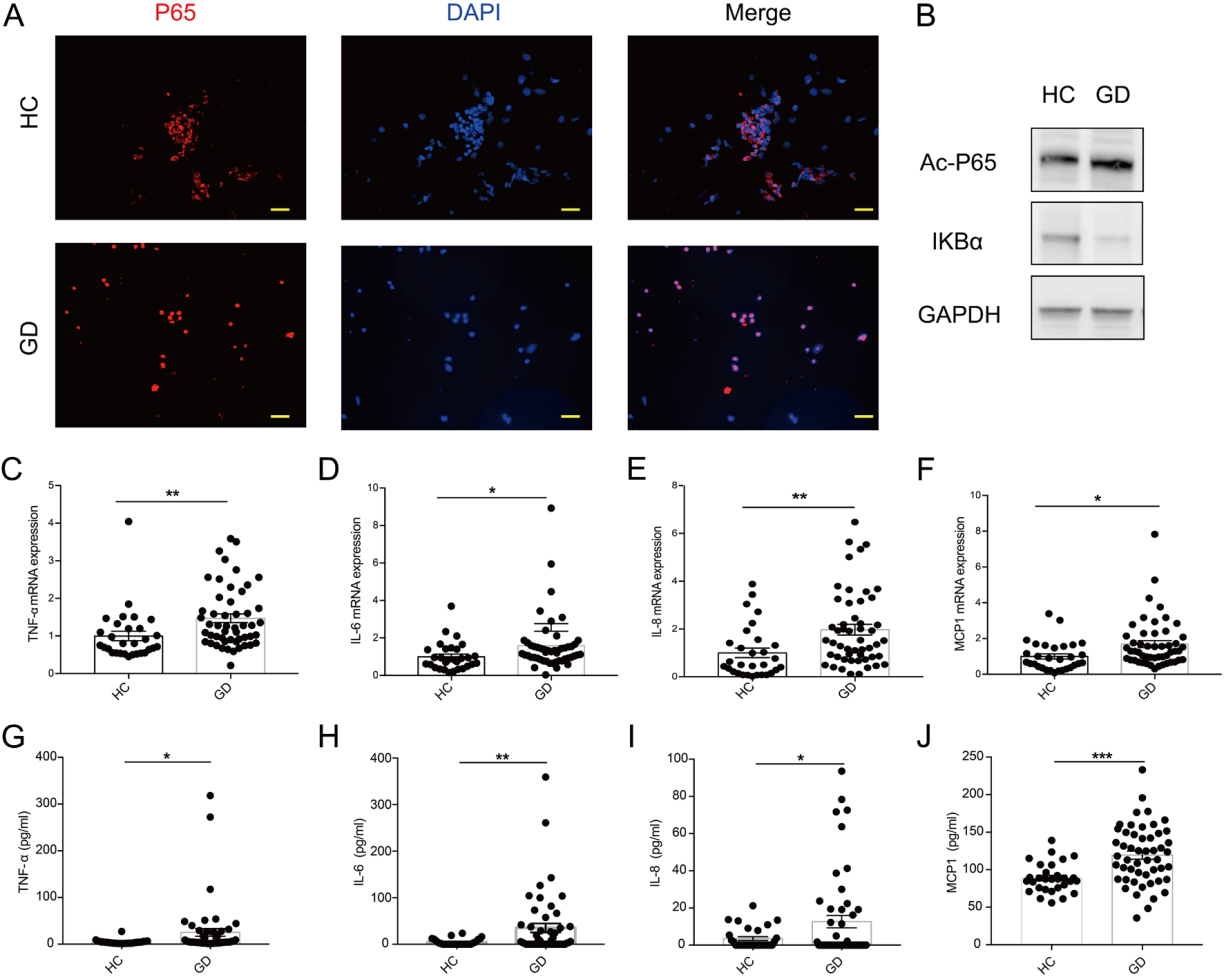
NF-κΒ signaling pathway is activated in GD patients. (A) Immunofluorescence of PBMCs with P65 (red) and DAPI (blue) (*n* = 5). Scale bars, 20 μm. (B) Western blotting analysis of key molecules of the NF-κB pathway in GD patient and HC PBMCs. (C–F) The mRNA expression of TNF-α (C), IL-6 (D), IL-8 (E) and MCP1 (F) was measured by qRT-PCR in GD patient (*n* = 51) and HC (*n* = 30) PBMCs. (G–J) The serum levels of TNF-α (G), IL-6 (H), IL-8 (I) and MCP1 (J) were measured by ELISA in the patients with GD (*n* = 51) and HCs (*n* = 30). GD, Graves’ disease; HC, healthy control. Data represent means ± s.e.m. *P < 0.05, **P < 0.01, ***P < 0.001.

The nuclear factor-κB family has been reported to be a substrate of SIRT1 and to be associated with inflammation. Much evidence has shown that the acetylation of P65 at lysine residues (particularly at 310) is required for the full transactivation of P65. Since SIRT1 expression was found to be decreased in GD patients, we examined whether the NF-κΒ pathway is activated in GD patients. Interestingly, P65-NF-κΒ was found to be located in the nucleus, which is evidence of active NF-κΒ signaling in PBMCs isolated from GD patients (Fig. 2A). Then, we used Western blotting to determine the main regulators of the NF-κΒ signaling pathway in GD patients and HCs. We found that the level of acetylated-P65 (ac-P65) was increased in GD patients. In addition, the expression of the IκB – an inhibitor of NF-κΒ – was decreased in GD patients (Fig. 2B, Supplementary Fig. 3). Finally, to further confirm whether the NF-κΒ pathway is involved in the pathogenesis of GD, we examined the levels of TNF-α, IL-6, IL-8 and MCP1 in the peripheral blood of the GD patients. The mRNA levels of *TNF-α* (Fig. 2C), *IL-6* (Fig. 2D), *IL-8* (Fig. 2E) and *MCP1* (Fig. 2F) in GD patients were significantly higher than those in HCs. Furthermore, GD patients showed much serum-higher protein levels of TNF-α (Fig. 2G), IL-6 (Fig. 2H), IL-8 (Fig. 2I) and MCP1 (Fig. 2J) than HCs. These findings indicate that the NF-κΒ signaling pathway appears to be activated in GD patients.

### Correlations between the levels of SIRT1 and NF-κB-associated proinflammatory cytokines and chemokine in peripheral blood

**Figure 3:**
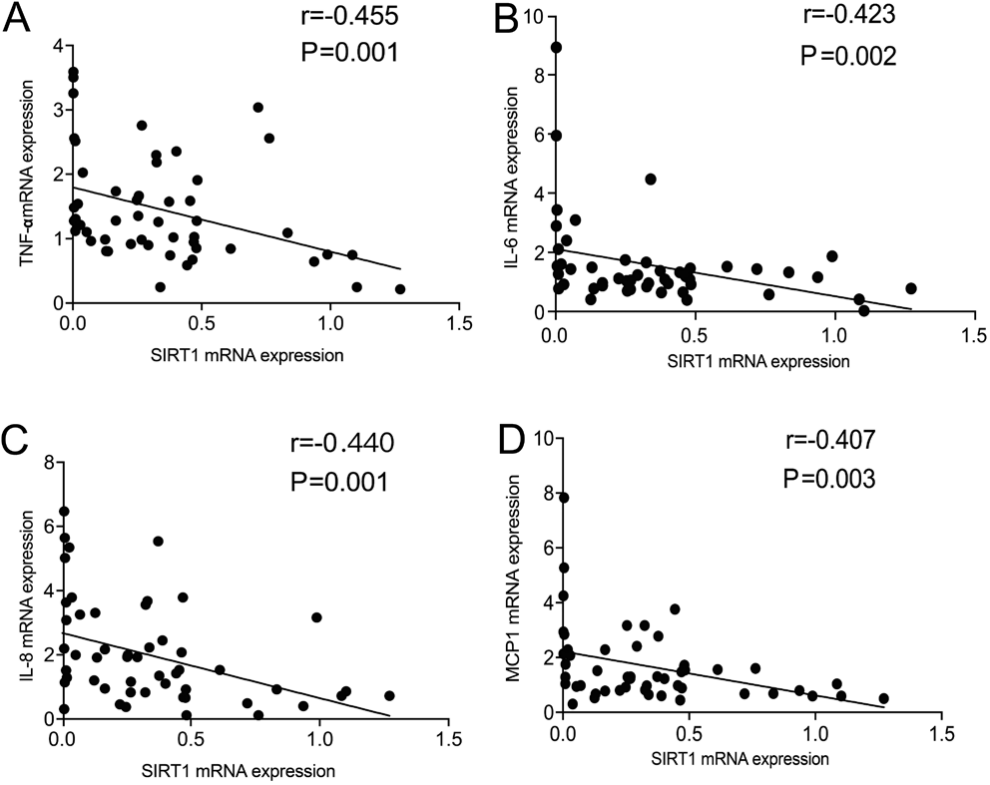
Correlations between the levels of SIRT1 and NF-κB-associated proinflammatory cytokines and chemokine in peripheral blood. (A–D) The correlation between mRNA expression of *TNF-α* (A), *IL-6* (B), *IL-8* (C), *MCP1* (D) and *SIRT1* in 51 GD patients. GD, Graves’ disease.

Previous research has reported that SIRT1 deacetylates P65, inhibiting its transactivation in non-small-cell lung cancer (NSCLC) cell lines (Yeung *et al.* 2004). To establish the relevance of these findings in GD patients, the relationships between the levels of SIRT1 and NF-κB-associated proinflammatory cytokines and chemokine in the peripheral blood were studied. An inverse correlation between *SIRT1* mRNA expression and *TNF-α* (*r* = −0.455, *P* = 0.001, Fig. 3A), *IL-6* (*r* = −0.423, *P* = 0.002, Fig. 3B), *IL-8* (*r* = −0.440, *P* = 0.001, Fig. 3C) and *MCP1* (*r* = −0.407, *P* = 0.003, Fig. 3D) mRNA expression was found in the GD patients. These results support the hypothesis that SIRT1 regulates the NF-κB pathway in GD patients.

### Increased SIRT1 expression and decreased NF-κB-associated proinflammatory factor expression in some patients with GD after treatment

**Figure 4:**
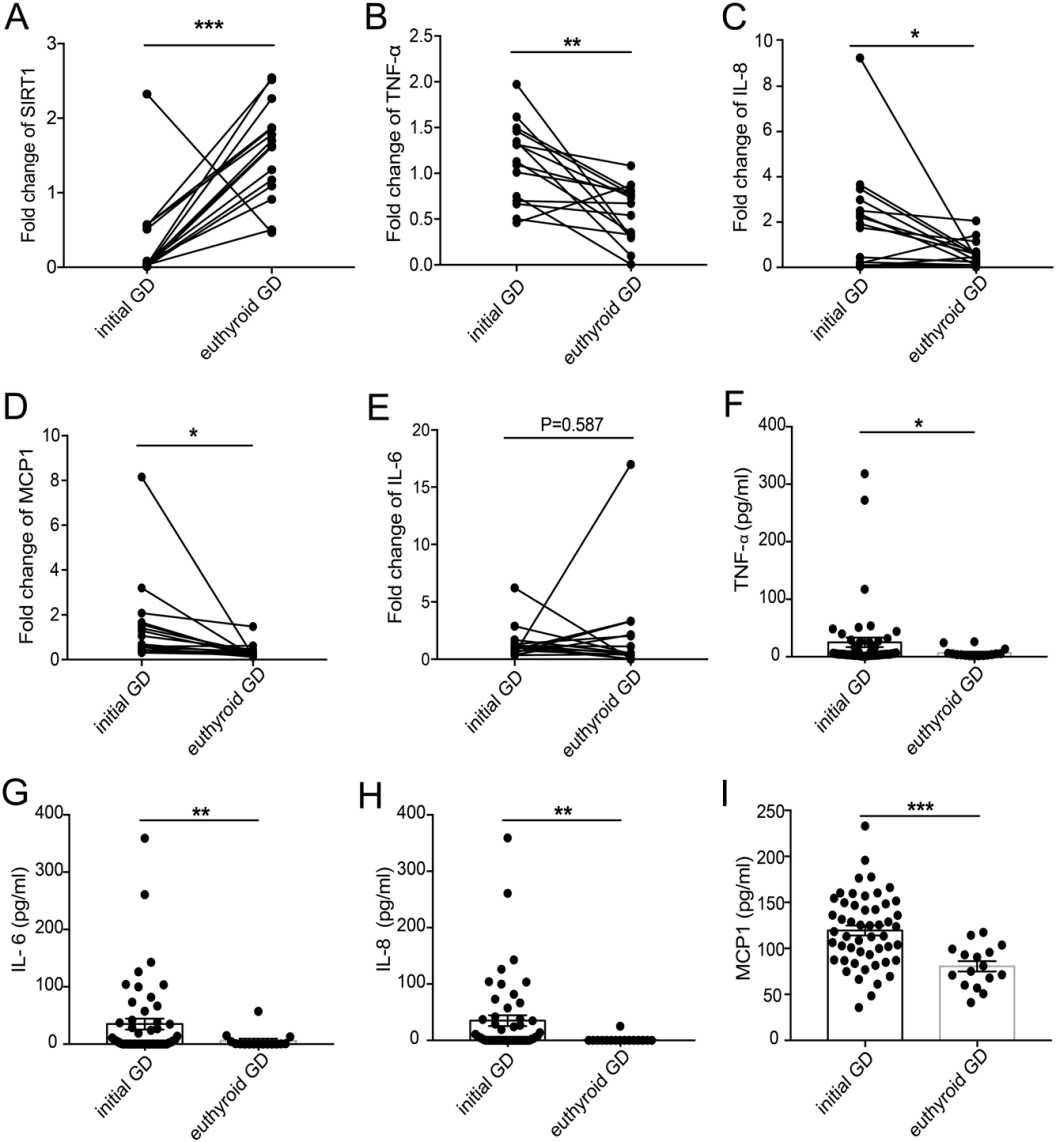
Increased SIRT1 expression and decreased NF-κB-associated proinflammatory factor expression in some patients with GD after treatment. (A) The mRNA expression change of *SIRT1* in PBMCs between pretherapy and posttreatment groups of GD patients. (B) The mRNA expression of *TNF-α* in patients with GD before and after treatment. (C) The mRNA expression change of *IL-8* in PBMCs between pretherapy and posttreatment groups of GD patients. (D) The mRNA expression of *MCP1* in patients with GD before and after treatment. (E) The mRNA expression change of *IL-6* in PBMCs between pretherapy and posttreatment groups of GD patients. (F) Serum levels of TNF-α in patients with GD before and after treatment. (G) Serum levels of IL-6 in GD patients treated with and without anti-thyroid drugs. (H) Serum levels of IL-8 in GD patients treated with and without anti-thyroid drugs. (I) Serum levels of MCP1 in patients with GD before and after treatment. GD, Graves’ disease. Data represent means ± s.e.m. *P < 0.05, **P < 0.01, ***P < 0.001.

We performed a follow-up analysis of 15 GD patients treated with methimazole therapy. After more than 3 months of treatment, the thyroid function clinical variables of the patients returned to normal levels. We found that the mRNA level of *SIRT1* was significantly increased (Fig. 4A) in euthyroid GD patients compared with initial GD patients. However, the expression of NF-κB-associated proinflammatory factors, such as *TNF-α* (Fig. 4B), *IL-8* (Fig. 4C), and *MCP1* (Fig. 4D), was markedly decreased. Although the mRNA level of *IL-6* was comparable between pretherapy and posttreatment samples, ten patients showed decreased expression of IL-6 (Fig. 4E). The protein levels of circulating TNF-α (Fig. 4F), IL-6 (Fig. 4G), IL-8 (Fig. 4H) and MCP1 (Fig. 4I) were found to be significantly reduced in euthyroid GD patients compared with initial GD patients.

### SIRT1 inhibition exacerbates the inflammatory response in PBMCs

**Figure 5:**
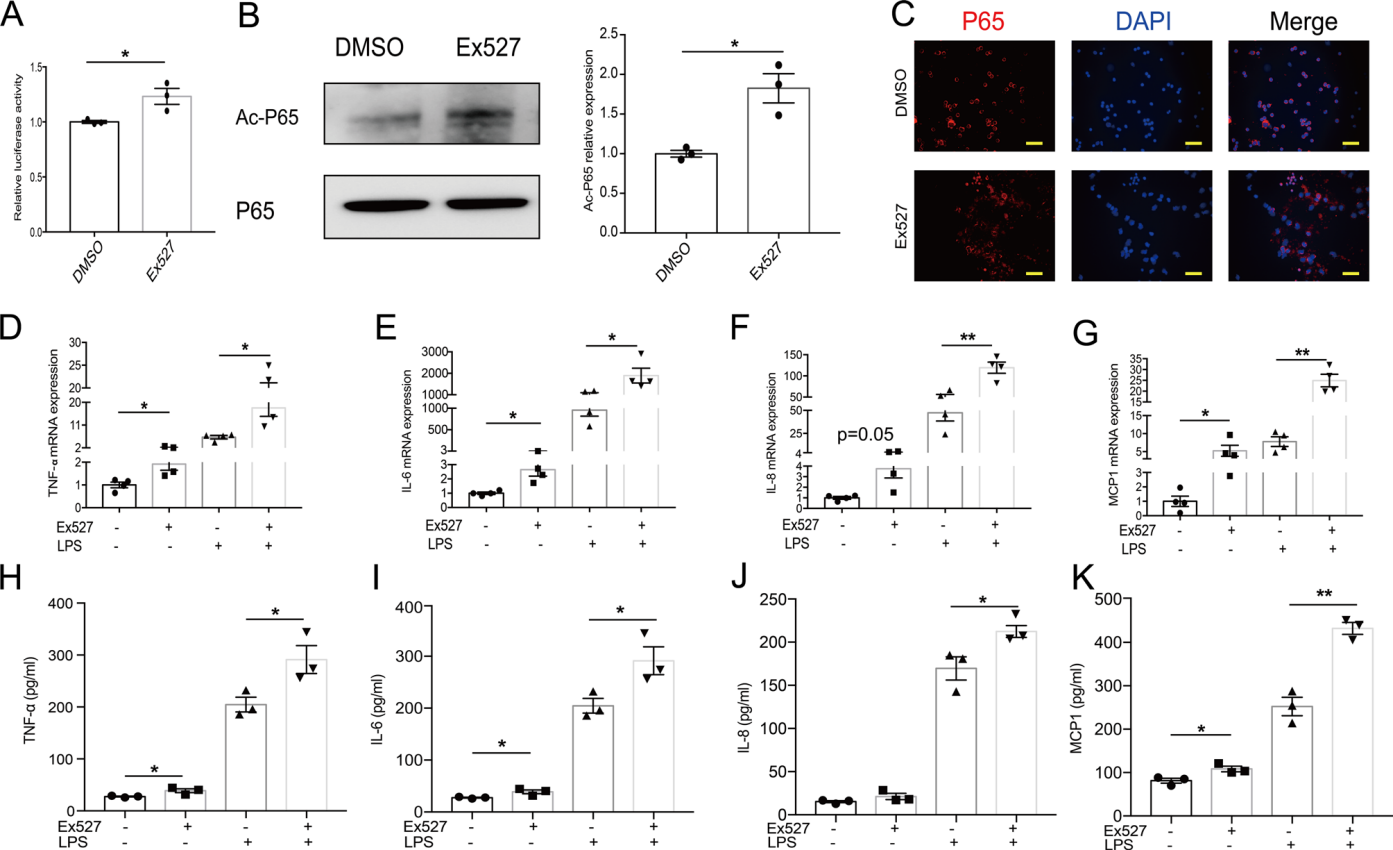
SIRT1 inhibition exacerbates the inflammatory response in PBMCs. (A) Transcriptional activities of NF-κB were analyzed by luciferase reporter assay (*n* = 3). (B–K) PBMCs were treated with DMSO or SIRT1-inhibitor Ex527 (40 μM) for 24 h. (B) The acetylation of P65 at K310 was determined by Western blotting (*n* = 3). Band intensities of Ac-P65 normalized for the corresponding P65 intensity were calculated (*n* = 3). (C) Immunostaining showed the nuclear translocation of P65 (red) when PBMCs were treated with Ex527 (*n* = 3). Scale bars, 20 μm. (D–G) PBMCs were stimulated with LPS (50 ng/mL) for the last 5-h incubation. The mRNA expression levels of *TNF-α* (D), *IL-6* (E), *IL-8* (F) and *MCP1* (G) were quantified by qRT-PCR (*n* = 4). (H–K) PBMCs treated with Ex527 were cultured with 50 ng/mL LPS for the last 5 h, and TNF-α (H), IL-6 (I), IL-8 (J) and MCP1 (K) concentrations in the culture supernatants were analyzed by ELISA (*n* = 3). Data represent means ± s.e.m. *P < 0.05, **P < 0.01.

As illustrated previously, the reduction in SIRT1 expression may play a pathogenic role in the development of GD. To explore the effects of SIRT1 on the inflammatory response, we studied whether the inhibition of SIRT1 in PBMCs affects the inflammatory response. Ex527, an inhibitor, was used. Compared with DMSO, Ex527 inhibited SIRT1 activity (Supplementary Fig. 4A). SIRT1 has been found to suppress the transcriptional activity of several transcription factors, such as P53. We thus asked whether the SIRT1 reduction induces NF-κB pathway activity via the transcriptional activity of NF-κB. Indeed, using a luciferase NF-κB reporter system, we found that treatment with Ex527 increased NF-κB transcriptional activity (Fig. 5A). Furthermore, P65 acetylation level were significantly increased in Ex527-treated PBMCs (Fig. 5B). The activation of the NF-κB signaling pathway was also confirmed by immunofluorescence (Fig. 5C). Interestingly, in PBMCs treated with Ex527, the inhibition of SIRT1 activity was accompanied by proinflammatory cytokine and chemokine expression that was higher than that in cells treated with DMSO, whether LPS was added or not (Fig. 5D–G). In agreement, the levels of TNF-α (Fig. 5H), IL-6 (Fig. 5I), IL-8 (Fig. 5J) and MCP1 (Fig. 5K) in the culture supernatant were also significantly increased after stimulation with Ex527, thus indicating that the inhibition of SIRT1 activity exacerbates the inflammatory response.

### SIRT1 activation alleviates the inflammatory response in GD patient PBMCs

**Figure 6:**
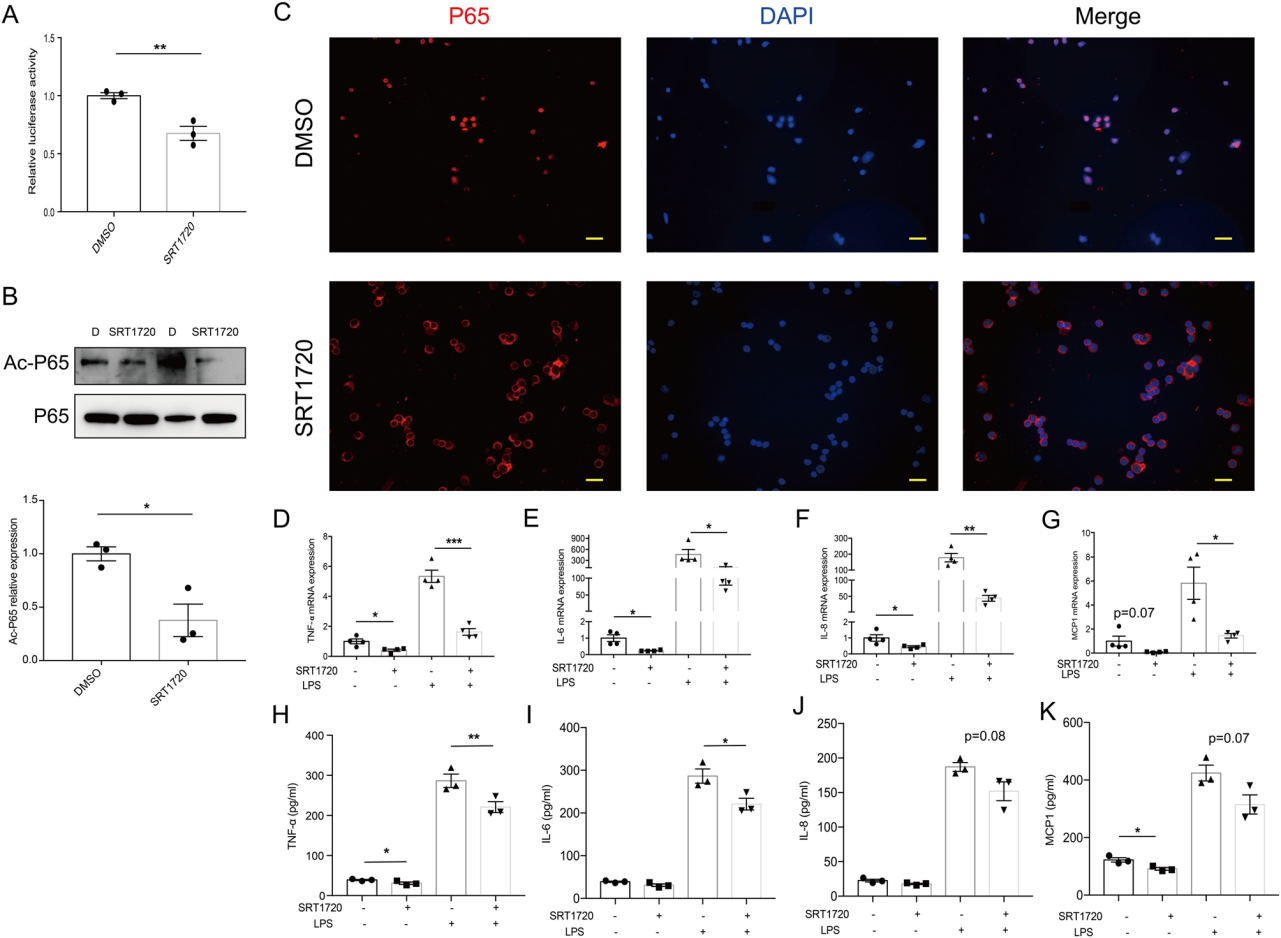
SIRT1 activation alleviates the inflammatory response in GD patient PBMCs. (A) Transcriptional activities of NF-κB were analyzed by luciferase reporter assay (*n* = 3). (B–K) PBMCs were treated with DMSO or SIRT1-activator SRT1720 (2 μM) for 24 h. (B) The acetylation of P65 at K310 was determined by Western blotting (*n* = 3). Band intensities of Ac-P65 normalized for the corresponding P65 intensity were calculated (*n* = 3). (C) Immunostaining showed the cytoplasm translocation of P65 (red) when GD patient PBMCs were treated with SRT1720 (*n* = 3). Scale bars, 20 μm. (D–G) GD patient PBMCs were stimulated with LPS (50 ng/mL) for the last 5-h incubation. The mRNA expression levels of *TNF-α* (D), *IL-6* (E), *IL-8* (F) and *MCP1* (G) were quantified by qRT-PCR (*n* = 4). (H–K) PBMCs treated with SRT1720 were cultured with 50 ng/mL LPS for the last 5 h, and TNF-α (H), IL-6 (I), IL-8 (J) and MCP1 (K) concentrations in the culture supernatants were analyzed by ELISA (*n* = 3). GD, Graves’ disease. Data represent means ± s.e.m. *P < 0.05, **P < 0.01, ***P < 0.001.

Next, we explored whether the activation of SIRT1 alleviates the inflammatory response in the PBMCs of GD patients. SRT1720, a putative SIRT1 activator, was used. Consequently, the SIRT1 activity in PBMCs treated with SRT1720 was observed to be significantly higher than that in PBMCs treated with DMSO (Supplementary Fig. 4B). SRT1720-treated cells showed decreased NF-κB transcriptional activity, as demonstrated by a luciferase reporter assay (Fig. 6A). Additionally, Western blotting analysis confirmed the decreased ac-P65 in GD patient PBMCs treated with SRT1720 (Fig. 6B), along with reduced NF-κB pathway activation confirmed by immunofluorescence (Fig. 6C). Moreover, SRT1720 treatment also resulted in the diminished expression of NF-κB target proinflammatory genes (Fig. 6D–G). In addition, the culture supernatant protein levels of TNF-α (Fig. 6H), IL-6 (Fig. 6I), IL-8 (Fig. 6J) and MCP1 (Fig. 6K) were also significantly decreased in the PBMCs treated with SRT1720. Taken together, our results show that the activation of SIRT1 is beneficial for alleviating the inflammatory response.

### Inhibition of SIRT1-induced inflammation is partially reduced by *P65* knockdown in PBMCs

**Figure 7:**
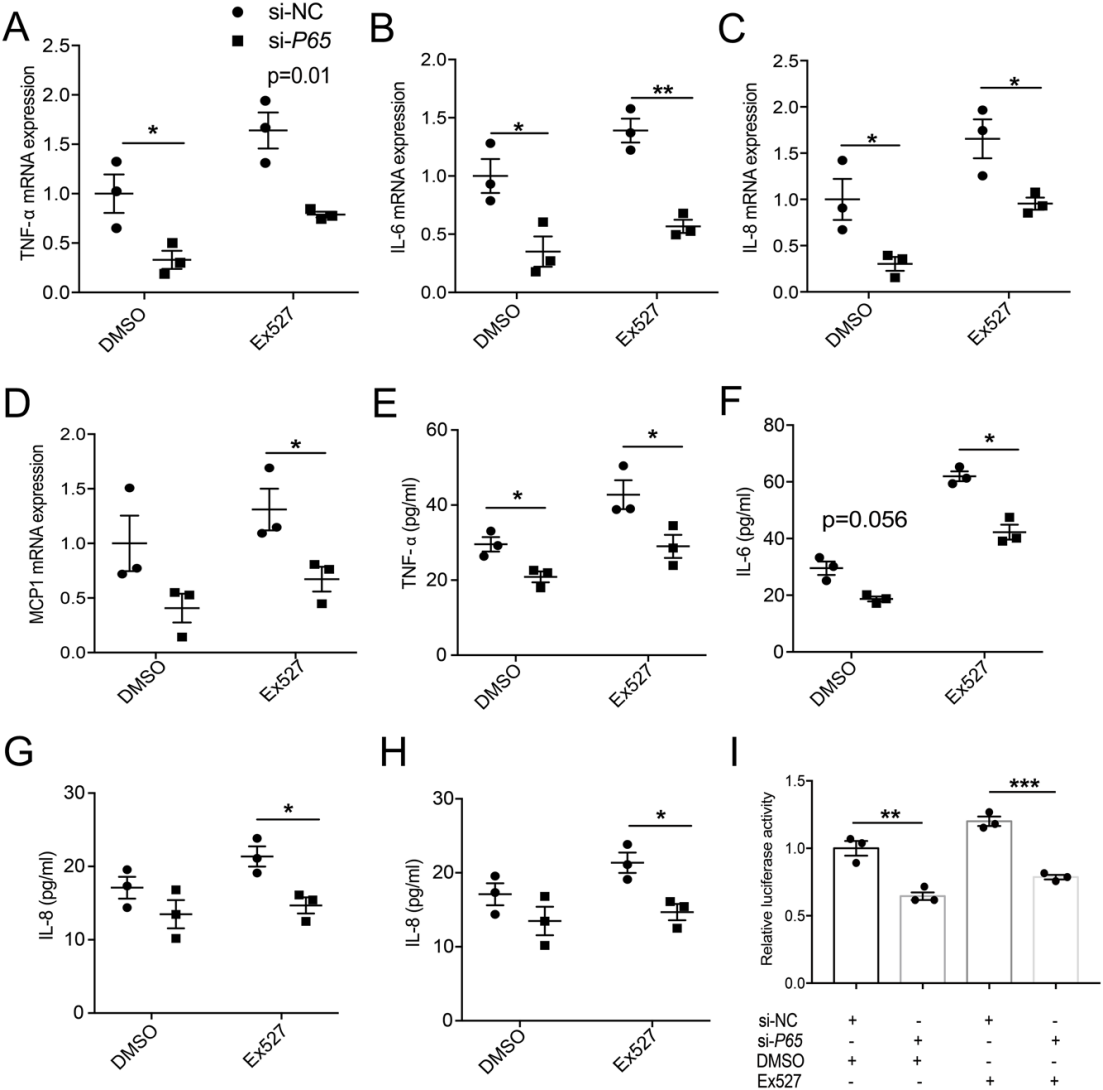
Inhibition of SIRT1-induced inflammation is partially reduced by *P65* knockdown in PBMCs. (A–H) PBMCs were transfected with *P65* siRNA or control siRNA and then the knockdown and control cells were treated with Ex527 (40 μM) or DMSO. The expression levels of *TNF-α* (A), *IL-6* (B), *IL-8* (C) and *MCP1* (D) were examined by qRT-PCR (*n* = 3). (E–H) TNF-α (E), IL-6 (F), IL-8 (G) and MCP1 (H) concentrations in the culture supernatants were analyzed by ELISA (*n* = 3). (I) HEK-293T cells were transiently transfected with *P65* siRNA or control siRNA. Ex527 and NF-κΒ promoter vectors were added 24 h before harvesting the cells, then transcriptional activities of NF-κB were analyzed by luciferase reporter assay (*n* = 3). Data represent means ± s.e.m. *P < 0.05, **P < 0.01, ***P < 0.001.

To further explore the relationship between SIRT1 and NF-κB, siRNA against *P65* was used. The efficiency of the siRNA transfection was confirmed by qRT-PCR (Supplementary Fig. 5). The mRNA levels of *TNF-α* (Fig. 7A), *IL-6* (Fig. 7B), *IL-8* (Fig. 7C) and *MCP1* (Fig. 7D) were reduced by the siRNA-mediated knockdown of *P65* in PBMCs. Moreover, the increased expression of these NF-κΒ-target inflammatory genes induced by Ex527 was blocked by *P65* knockdown (Fig. 7A–D). Consistent with these results, the levels of TNF-α (Fig. 7E), IL-6 (Fig. 7F), IL-8 (Fig. 7G) and MCP1 (Fig. 7H) in the culture supernatant were also significantly decreased in the presence of *P65* siRNA. Furthermore, although the protein levels of TNF-α (Fig. 7E), IL-6 (Fig. 7F), IL-8 (Fig. 7G) and MCP1 (Fig. 7H) were increased by Ex527 treatment, these changes were blocked by the transfection of si-*P65*. The siRNA-mediated knockdown of *P65* in HEK 293T cells decreased the transcriptional activity of NF-κB, regardless of whether Ex527 was added, as demonstrated by a luciferase reporter assay (Fig. 7I). These results suggest that SIRT1 inhibits the inflammatory response via NF-κB.

## Discussion

GD is one of the most frequent diseases among autoimmune disorders, and in iodine-sufficient areas, it accounts for 70–80% of all cases of thyrotoxicosis (Abraham-Nordling *et al.* 2011). Although anti-thyroid drugs can be used to restore euthyroidism in all patients, these drugs often cause negative side effects in patients (David 2005, De Groot *et al.* 2012).

The results of the current study demonstrate for the first time that SIRT1 is an important regulator involved in the development of GD via the NF-κΒ pathway. This is suggested by several lines of evidence. First, SIRT1 is expressed in CD4+ T, CD8+T, B-lymphocytes and monocytes which are the main composition of PBMCs, and SIRT1 expression is downregulated in the PBMCs of GD patients, especially in CD14+ monocytes. Second, NF-κΒ pathway activation is observed in GD patients. Third, low SIRT1 expression is associated with GD patient clinical variables, and significant correlations between *IL-6*, *IL-8*, *TNF-α*, *MCP1* and *SIRT1* are present at the mRNA levels in GD patients. Fourth, the inhibition of SIRT1 exacerbates the inflammatory response in healthy control PBMCs, whereas the activation of SIRT1 improves the inflammatory response in GD patient PBMCs. Finally, our findings suggest that P65 is a direct target of SIRT1 and that SIRT1 mediates the inflammatory response via NF-κB in GD.

SIRT1 regulates the functions of several important transcription factors with anti-inflammatory effects (Park *et al.* 2016, Hah *et al.* 2014). A large body of evidence suggests that SIRT1 plays a major role in various diseases, such as rheumatoid arthritis (Li *et al.* 2018), atherosclerosis (Sosnowska *et al.* 2017), insulin sensitivity (Hui *et al.* 2017) and kidney disease (He *et al.* 2010, Hasegawa *et al.* 2010). However, whether PBMCs SIRT1 is involved in the pathogenesis of GD remains unknown. In the present study, qRT-PCR, Western blotting and immunofluorescence assays confirmed the downregulated expression of SIRT1. Further studies confirmed that SIRT1 expression strongly correlated with clinical GD parameters, including FT3 and FT4, especially TRAb levels. A limitation should also be mentioned. Although higher TRAb levels are often found in those GD patients that also have orbitopathy, in our 51 patients with Graves’ disease, there is nobody suffering thyroid-associated ophthalmopathy, so we cannot analyze the association between SIRT1 levels and orbitopathy. Further recruitment of GD patients with orbitopathy will be needed to explore the association between SIRT1 levels and thyroid-associated orbitopathy. After MMI treatment, *SIRT1* expression was upregulated in euthyroid patients. In a previous study, the mRNA level of *SIRT1* remained unchanged (Yan *et al.* 2015); however, we think this difference in our study may be due to our stricter enrollment criteria, and subjects with other factors which might influence SIRT1 expression have been excluded. Furthermore, in their article, SIRT1 mRNA level showed a tendency of decrease in PBMCs from GD patients compared to healthy controls, yet not to statistical significance (Yan *et al.* 2015). Due to their small cohort size, we have enlarged the healthy control and GD patient cohort size.

Recent evidence suggests that transcription factor activation is regulated not only by protein phosphorylation, but also by protein acetylation. SIRT1 exerts biological effects not only through the deacetylation of histones, but also through the deacetylation of various transcription factors, including P53, FOXO, P65, STAT3, PGC1α, and PPAR-γ (Nakagawa & Guarente 2011), thereby leading to transcriptional repression. SIRT1 could exert anti-inflammatory effects through the inhibition of the NF-κB pathway. It has been shown that the duration of nuclear NF-κB activity is highly regulated by reversible acetylation (Senftleben *et al.* 2001) and that SIRT1 inhibits the NF-κB signaling pathway through the deacetylation of P65 (Yang *et al.* 2010). A well-recognized function of NF-κB is the regulation of inflammatory responses. In addition to mediating the induction of the expression of various proinflammatory genes in innate immune cells, NF-κB regulates the activation, differentiation and effector function of inflammatory T cells (Lawrence 2009, Tak & Firestein 2001). Recent evidence suggests that NF-κB also plays a role in regulating the activation of inflammasomes (Zhong *et al.* 2016, Afonina *et al.* 2017). In the present study, we found that P65 translocated in the nucleus in GD patients, whereas P65 was sequestered in the cytoplasm in HCs. Western blotting confirmed that the level of the NF-κB inhibitor IκΒα was decreased in GD patients compared with HCs, thus causing the rapid and transient nuclear translocation of P65 and subsequent activation of proinflammatory cytokines and chemokines. The increases in IL-6, IL-8, TNF-α and MCP1 were confirmed in GD patients at both the mRNA and protein levels.

To establish whether SIRT1 regulates NF-κΒ in GD patients, we further confirmed the association between SIRT1 expression and NF-κB-target proinflammatory cytokine and chemokine expression. *SIRT1* expression was inversely correlated with *IL-6*, *IL-8*, *TNF-α* and *MCP1* expression. Furthermore, SIRT1 expression levels increased in euthyroid GD patients, indicating that the suppression of SIRT1 expression and activity in PBMCs might be a prerequisite for increased susceptibility to the development of inflammation or an initial change in the pathogenesis of GD. Next, we treated healthy control PBMCs with the SIRT1 inhibitor Ex527 and the PBMCs of GD patients with the SIRT1 activator SRT1720. We found that Ex527 treatment upregulated P65 acetylation at K310 and increased the nuclear translocation of NF-κB P65, which is consistent with previous reports (Yeung *et al.* 2004, Ghisays *et al.* 2015). In addition, the expression of NF-κB-regulated proinflammatory cytokines and chemokines increased in response to Ex527 treatment. In contrast, SRT1720 treatment suppressed the production of proinflammatory cytokines and chemokines and nuclear translocation of P65, returning it to the cytoplasm. Furthermore, P65 acetylation was decreased. Importantly, the results from the siRNA-mediated knockdown of *P65* together with the Ex527 treatment further confirmed the conclusion that SIRT1 regulates the inflammatory response via the NF-κB pathway. These results are also consistent with those of Park *et al.* who showed that the PMA-induced transcriptional activation and secretion of NF-κB-regulated proinflammatory cytokines (TNF-α, IL-1β and IL-6) are suppressed by resveratrol treatment. Furthermore, the suppression of NF-κB transcriptional activity is associated with the greater reduction in TNF-α, IL-1β and IL-6 mRNA and protein levels in SIRT1 transgenic mice compared to control C57BL/6 mice (Park *et al.* 2016).

In summary, our results show that SIRT1 plays a role in the pathogenesis of GD by regulating the NF-κB pathway and that SIRT1 probably acts as a negative regulator of the inflammatory processes associated with GD. We conclude that SIRT1 modulation offers a promising strategy for the treatment of GD inflammation.

## Supplementary Material

Supplementary Table 1. The clinical characteristics of patients with Hashimoto thyroiditis and healthy controls.

Supplementary Table 2. Changes in clinical characteristics in patients with Graves’ disease at baseline and following therapy of methimazole.

Supplementary Table 3. Primers sequences used for qRT-PCR in the study.

Figure S1. Images of immunofluorescence on SIRT1 positive on the type of PBMCs.

Figure S2. The mRNA expression of SIRT1 was decreased in patients with Hashimoto thyroiditis.

Figure S3. Western blotting analysis of key molecules of the NF-κB pathway in GD patient and HC PBMCs.

Figure S4. SIRT1 activity was determined after treatment.

Figure S5. Silencing effects of si-p65 were examined by qRT-PCR.

## Declaration of interest

The authors declare that there is no conflict of interest that could be perceived as prejudicing the impartiality of the research reported.

## Funding

This work was supported by the National Natural Sciences Foundation of China Grants (81873637, 81570707, 81800693).

## Author contribution statement

S W, Q Y and D L designed the experiments. Q Y, L S, Y Q, Y P, Q W and Z J performed the experiments. D S, L Y, G N and W W provided helpful discussions. S W, Q Y, and L S analyzed data and wrote the paper. S W was responsible for research supervision, coordination and strategy.

## Acknowledgements

The authors thank Canqi Cui and Tingting Li for helpful discussions.
