# Supplementary material for: Decreased SIRT1 expression in the peripheral blood of patients with Graves’ disease
Source: J Endocrinol. 2020 Jun 2;246(2):161–73. doi: 10.1530/JOE-19-0501 (PMC7354706; doi:10.1530/JOE-19-0501)
Supplement: Supplementary Table 1. The clinical characteristics of patients with Hashimoto thyroiditis and healthy controls. [file supplementary_table_1.pdf]

Supplementary Table 1. The clinical characteristics of patients with Hashimoto thyroiditis and healthy controls.

Variable	HC	HT	Normal Range
No.	30	17	-
Age (years)	35±12	38±14	-
Gender (M/F)	8/22	1/16	-
FT3 (pmol/L)	4.2±0.5	4.3±0.7	2.63-5.70
FT4 (pmol/L)	12.6±2.8	12.8±3.6	9.01-19.04
TSH (μIU/mL)	2.19±0.85	3.16±2.64	0.3500-4.9400
TRAb (IU/L)	-	-	< 1.75
TPOAb (IU/ml)	1.30±1.71	336.65±356.21	< 5.61
TGAb (IU/ml)	0.79±1.19	494.51±340.15	< 4.11

Data are expressed as mean ± standard deviation according to the distribution.

M, male; F, female. "-" represents that the experiment was not performed, or the data are not available. HT, Hashimoto thyroiditis; HC, healthy control.
