# Supplementary material for: Decreased SIRT1 expression in the peripheral blood of patients with Graves’ disease
Source: J Endocrinol. 2020 Jun 2;246(2):161–73. doi: 10.1530/JOE-19-0501 (PMC7354706; doi:10.1530/JOE-19-0501)
Supplement: Supplementary Table 2. Changes in clinical characteristics in patients with Graves’ disease at baseline and following therapy of methimazole. [file supplementary_table_2.pdf]

Supplementary Table 2. Changes in clinical characteristics in patients with Graves' disease at baseline and following therapy of methimazole.

**Variable**	**Initial GD**	**Euthyroid GD**	***P***
No.(M/F)	15(1/14)	15(1/14)	
Age	37±15	37±15	
FT3 (pmol/L)	28.8±16.6	4.0±0.7	<0.001
FT4 (pmol/L)	43.4±14.8	12.8±2.2	<0.001
TSH (μIU/mL)	0.001±0.0002	1.49±0.47	0.006
TRAb (IU/L)	16.2±9.7	6.7±5.1	<0.001
TPOAb(IU/ml)	463.1±406.5	302.0±387.1	0.017
TGAb (IU/ml)	119.0±141.5	36.2±75.8	0.011

Data are expressed as mean ± standard deviation according to the distribution.

M, male; F, female.
