# Supplementary material for: Decreased SIRT1 expression in the peripheral blood of patients with Graves’ disease
Source: J Endocrinol. 2020 Jun 2;246(2):161–73. doi: 10.1530/JOE-19-0501 (PMC7354706; doi:10.1530/JOE-19-0501)
Supplement: Supplementary Table 3. Primers sequences used for qRT-PCR in the study. [file supplementary_table_3.pdf]

Supplementary Table 3. Primers sequences used for qRT-PCR in the study.

	Forward primer	Reverse primer
SIRT1	TAGGCGGCTTGATGGTAA	ATGGGTTCTTCTAAACTTGG
MCP1	TCAAACTGAAGCTCGCACTC	ATTGATTGCATCTGGCTGAG
IL-6	GACAGCCACTCACCTCTTCA	CCTCTTTGCTGCTTTCACAC
TNF-α	TTTGATCCCTGACATCTGGA	GGCCTAAGGTCCACTTGTGT
IL-8	AGGACAAGAGCCAGGAAGAA	ACTGCACCTTCACACAGAGC
P65	AGCACAGATACCACCAAGAC	TGGTCCCGTGAAATACACCT
GADPH	GGAGCGAGATCCCTCCAAAAT	GGCTGTTGTCATACTTCTCATGG
