# Supplementary figures and images for: Decreased SIRT1 expression in the peripheral blood of patients with Graves’ disease
Source: J Endocrinol. 2020 Jun 2;246(2):161–73. doi: 10.1530/JOE-19-0501 (PMC7354706; doi:10.1530/JOE-19-0501)

**A**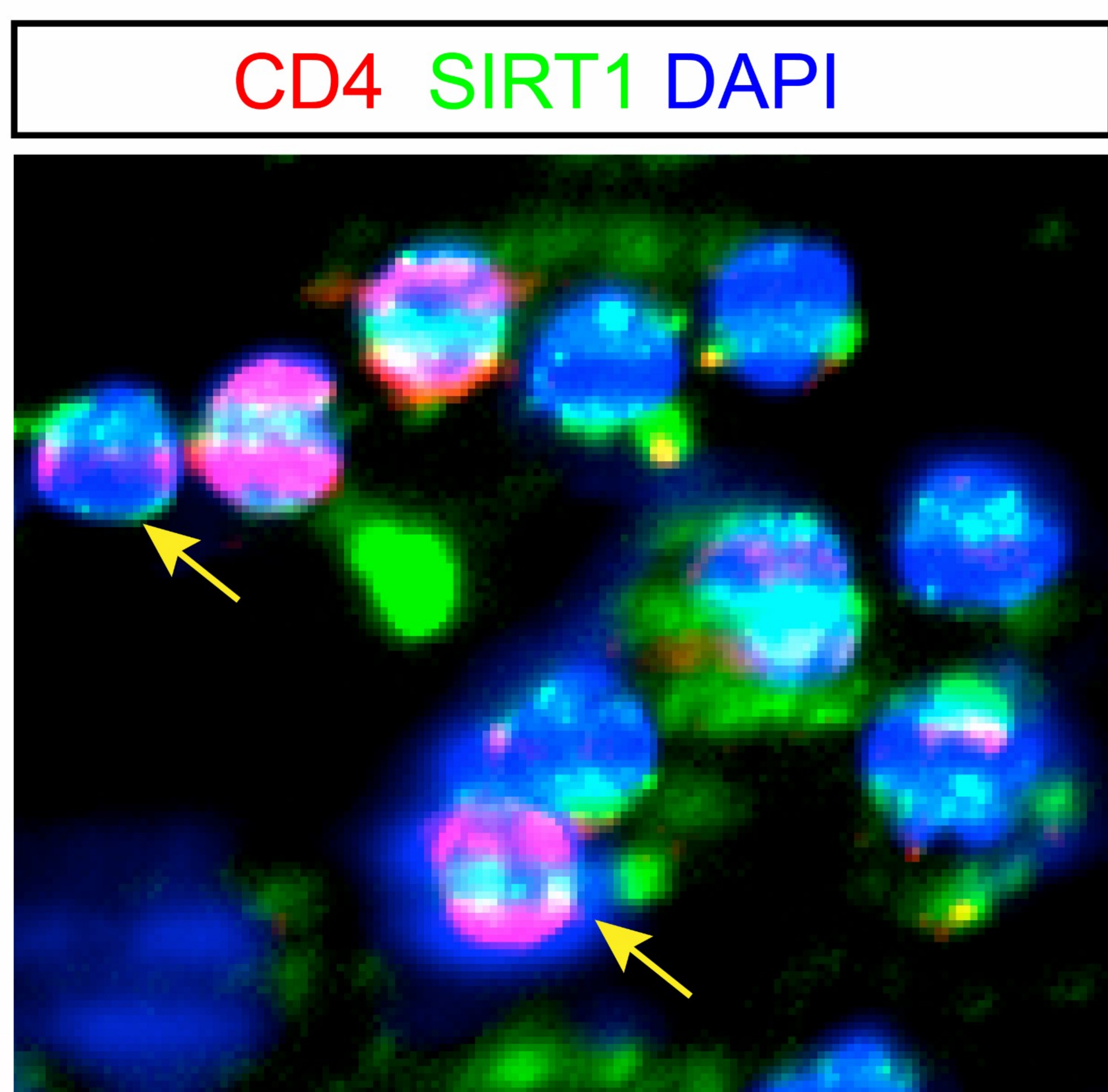**B**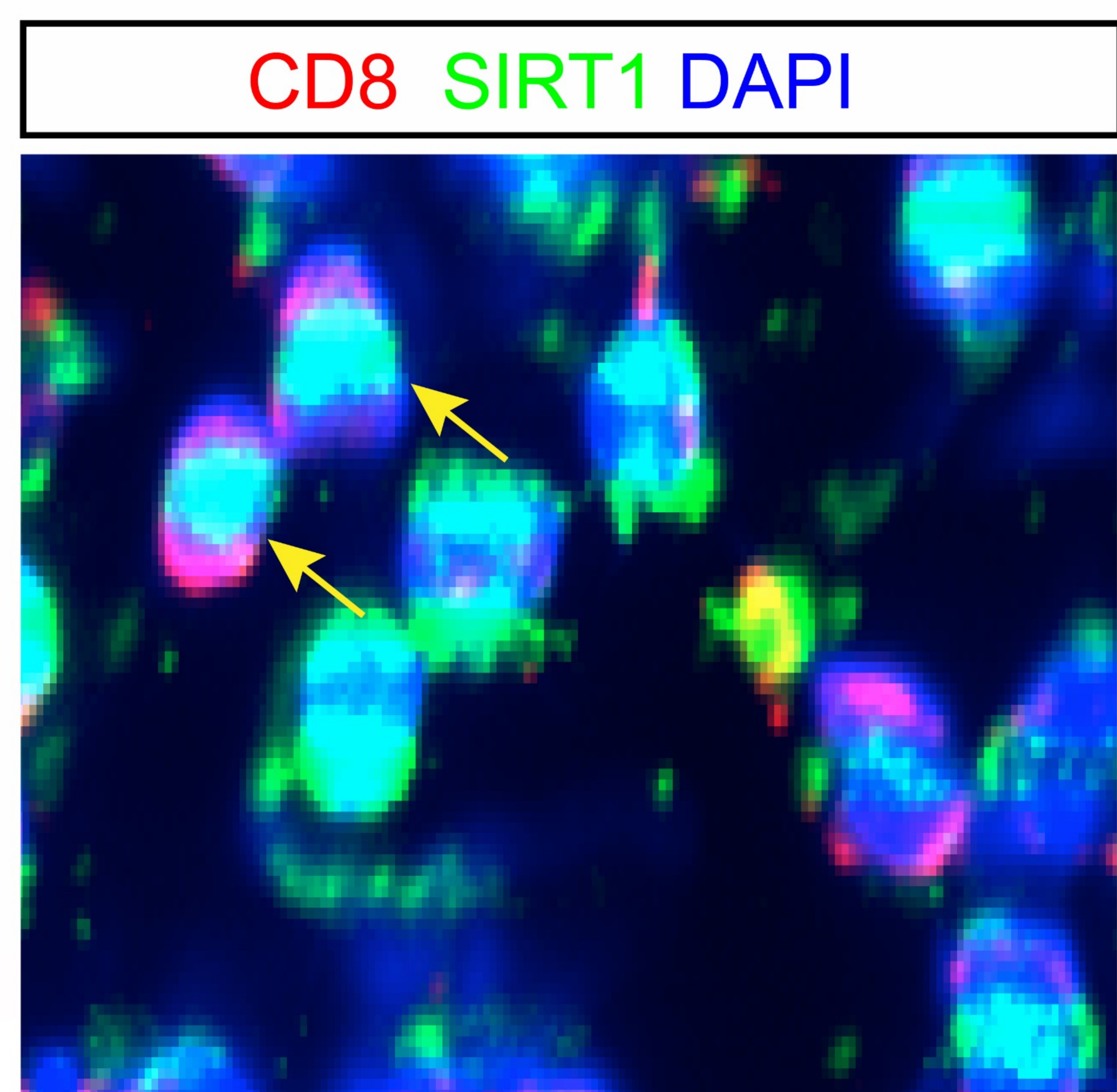**C**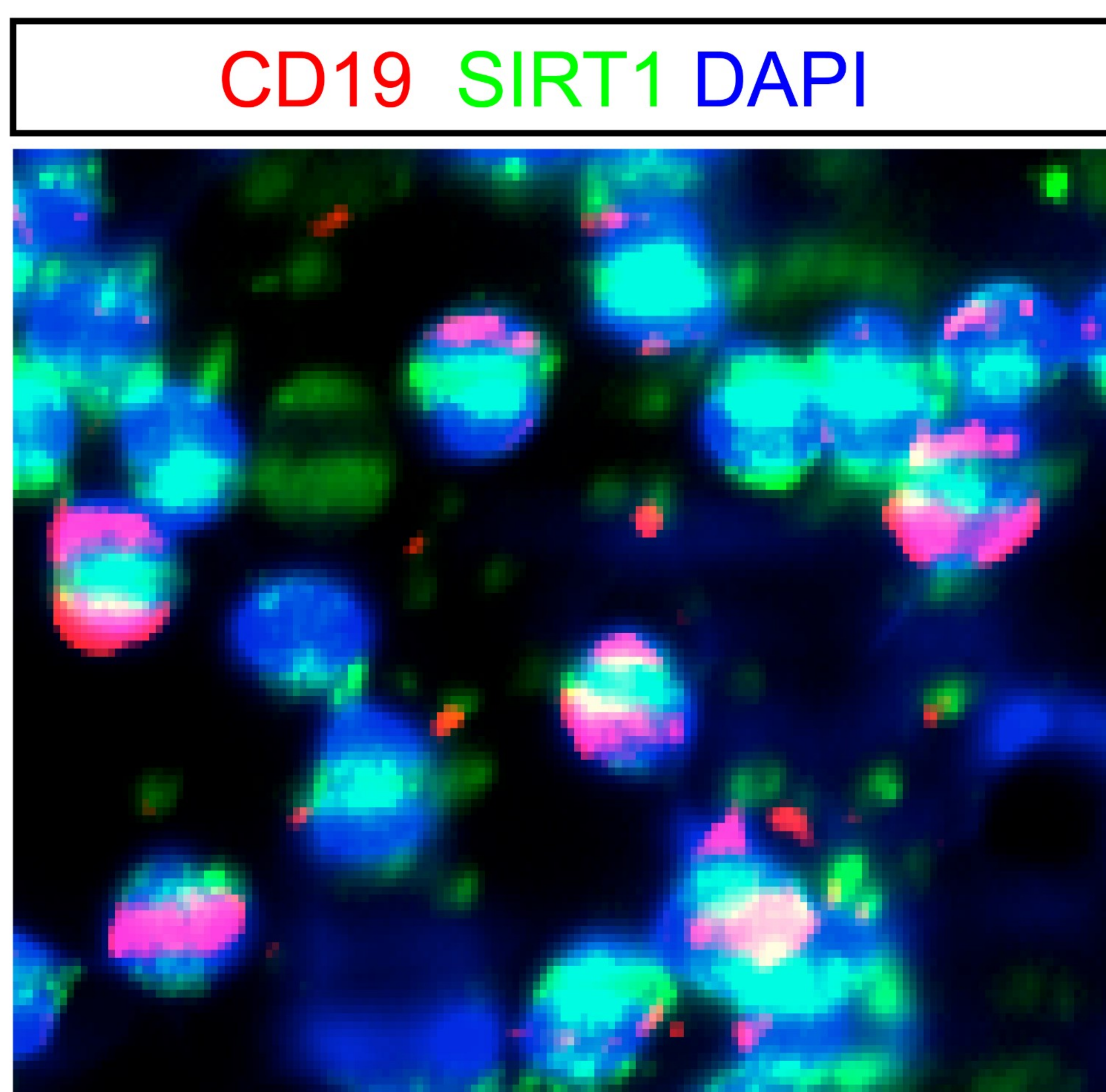**D**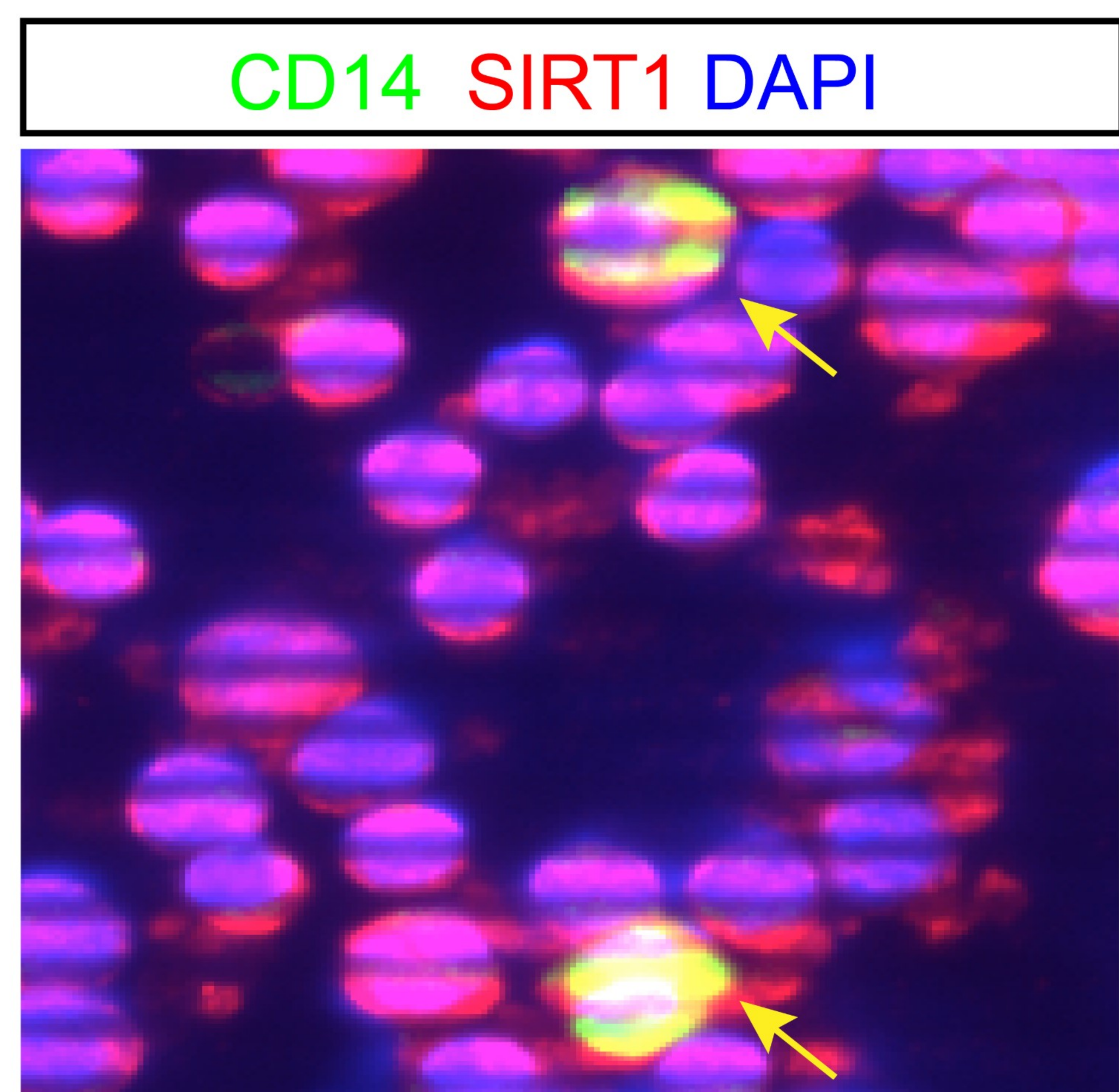**E**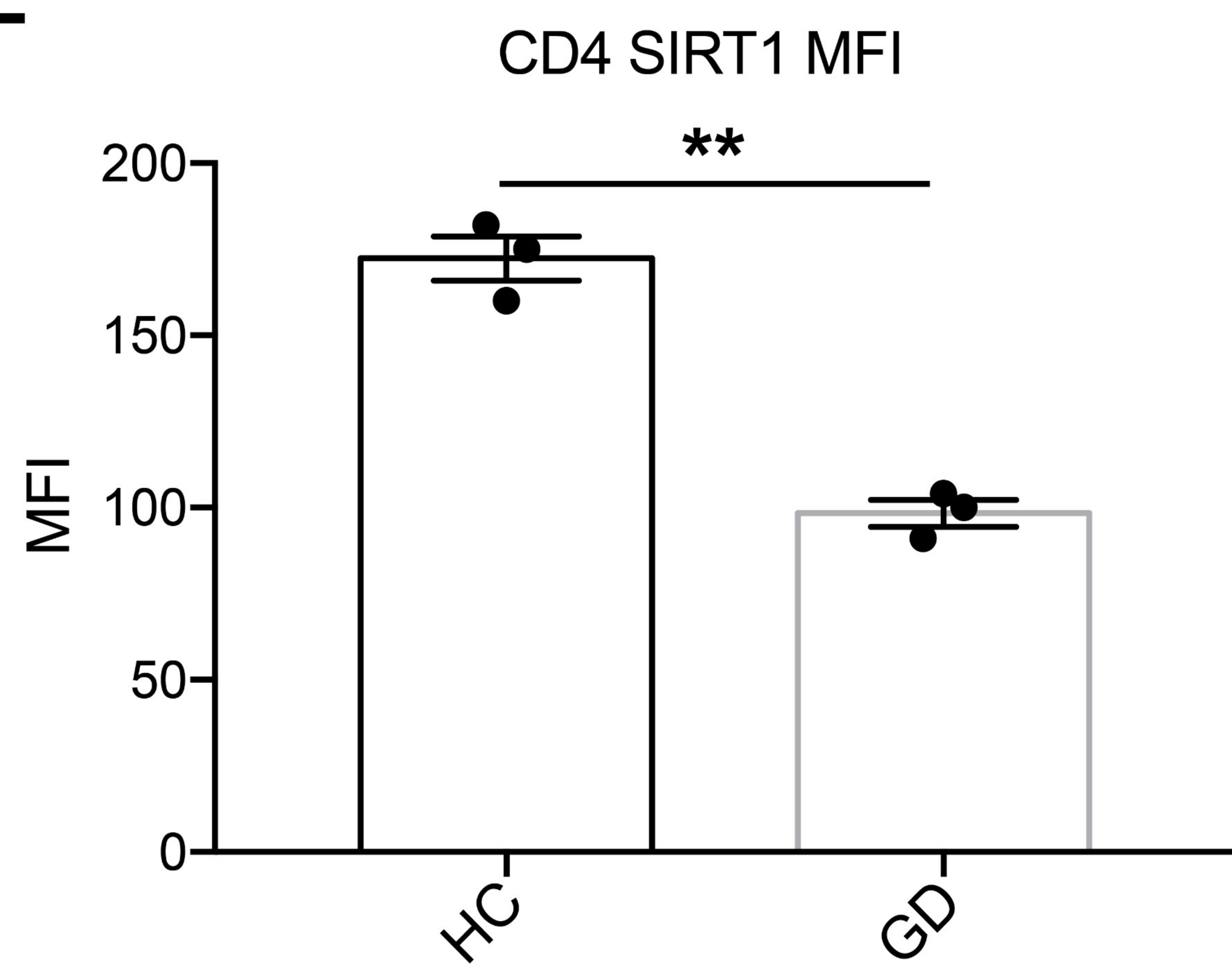**G**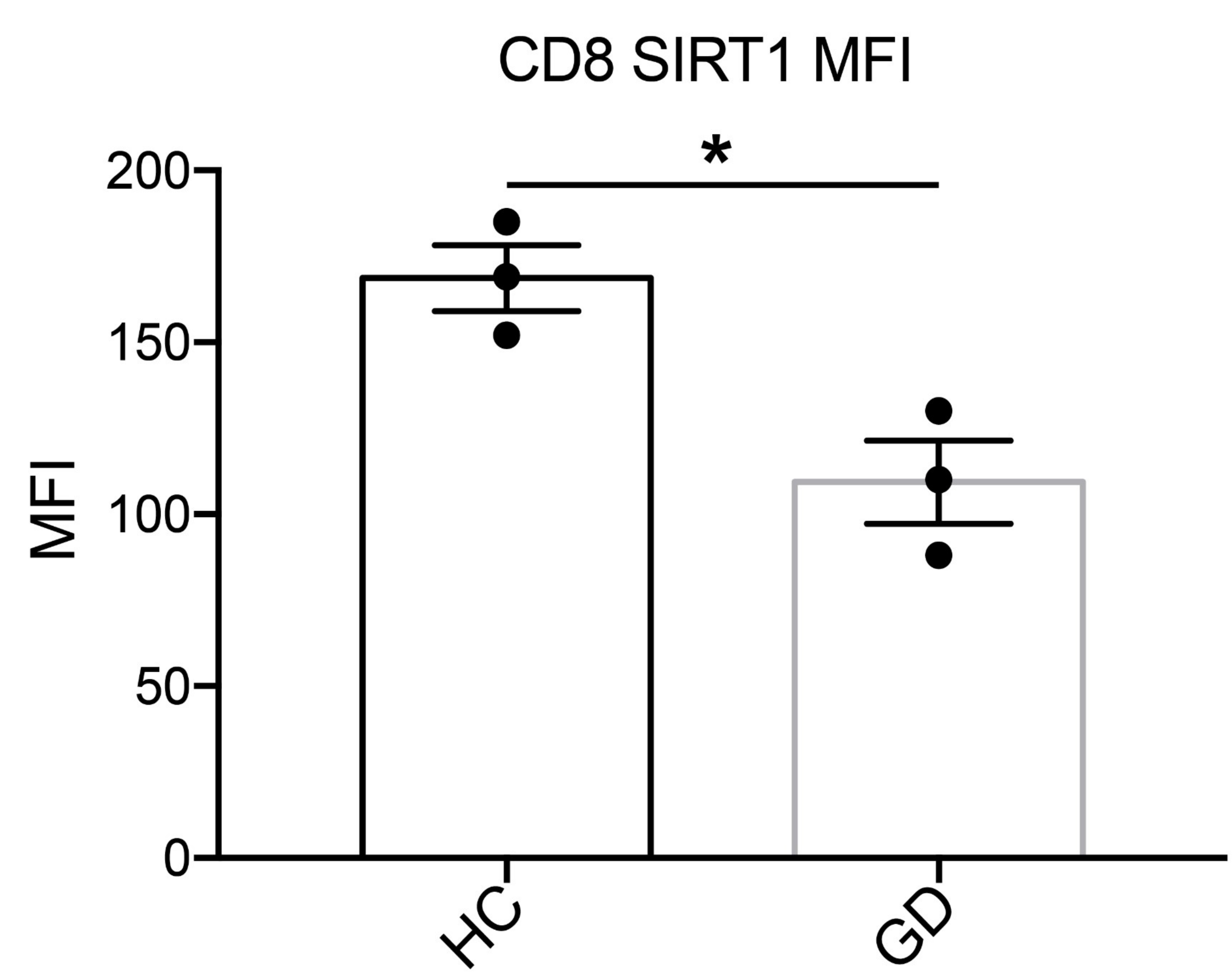**G**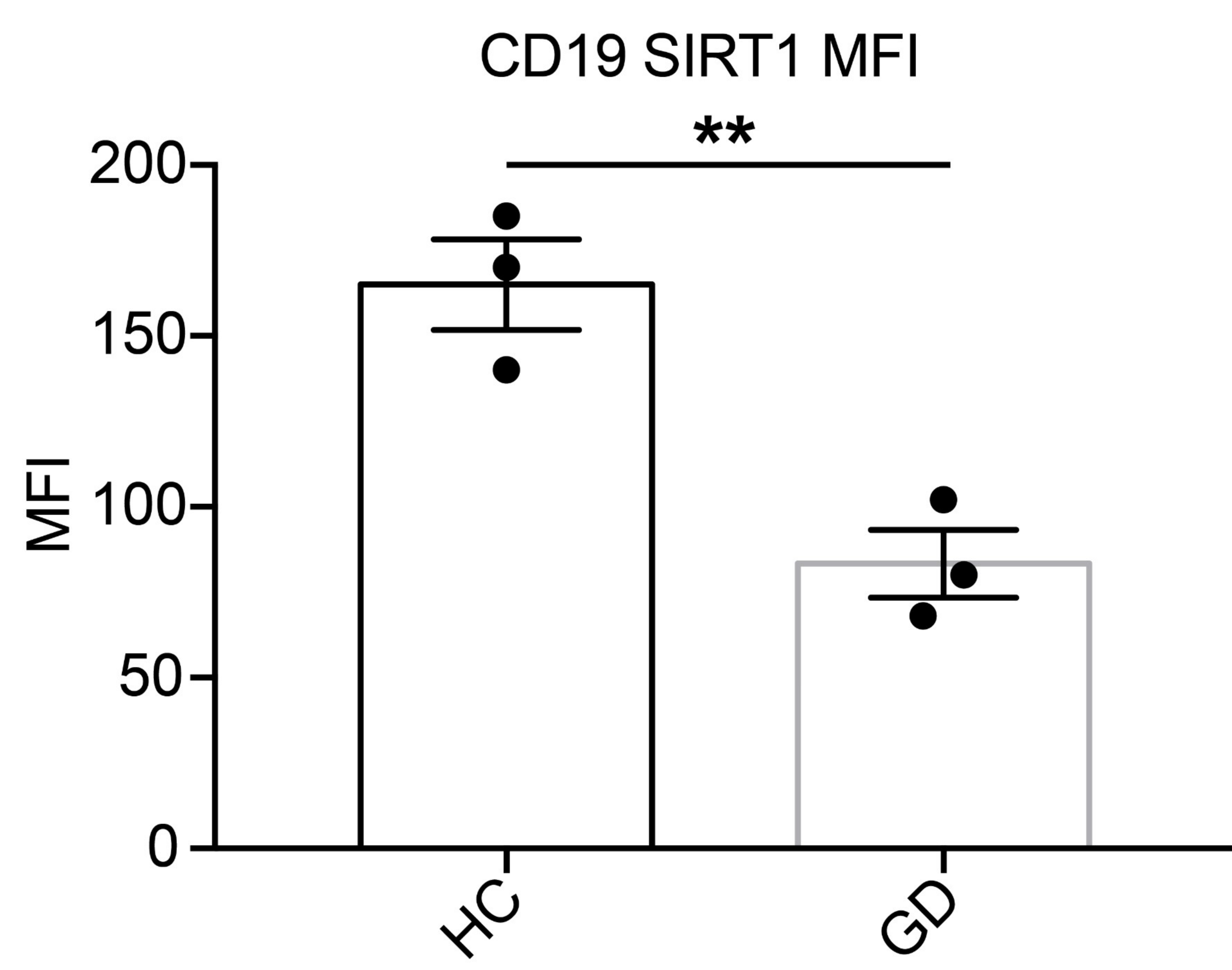**H**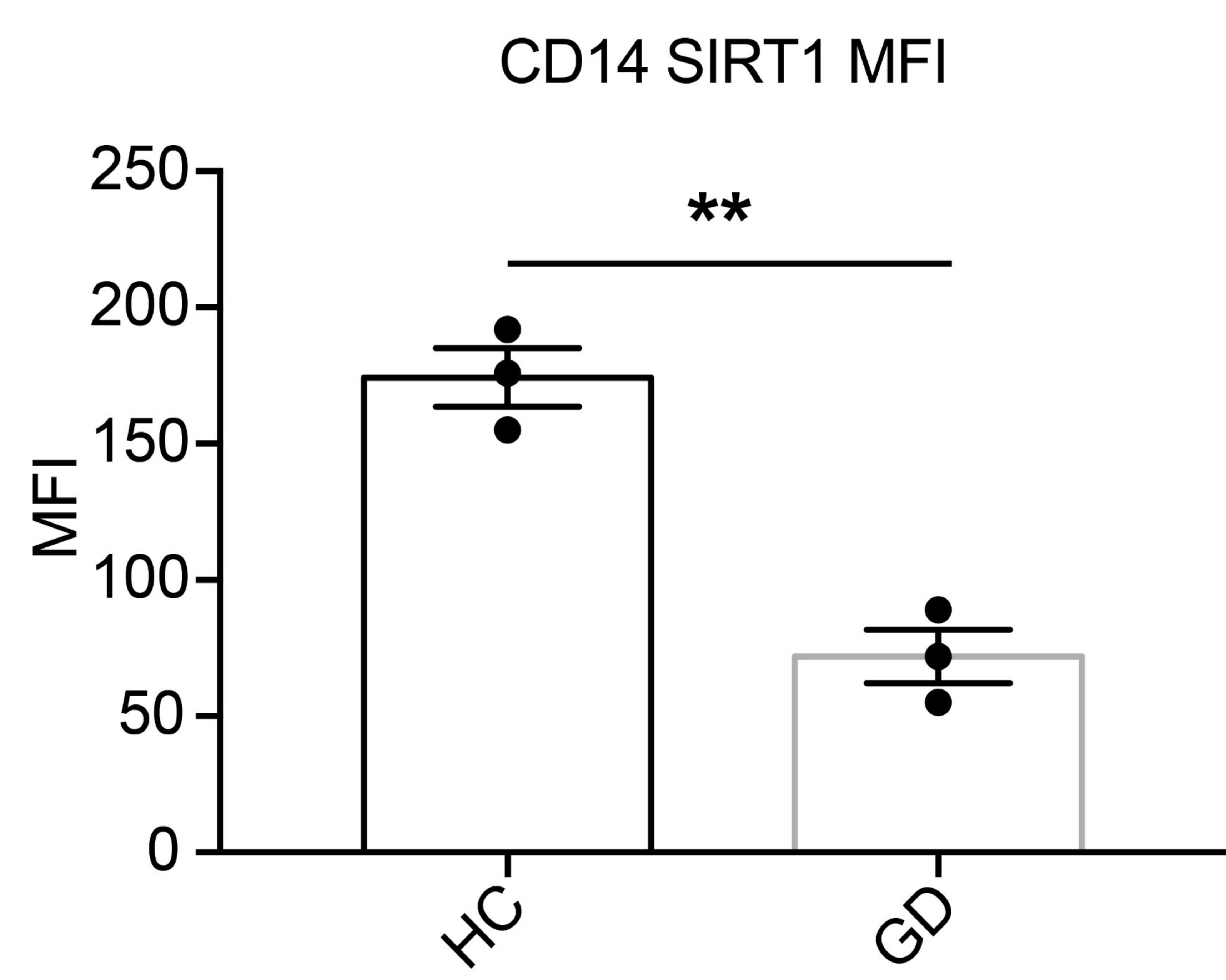

Supplement: Figure S1. Images of immunofluorescence on SIRT1 positive on the type of PBMCs. [file supplementary_figure_1.pdf]

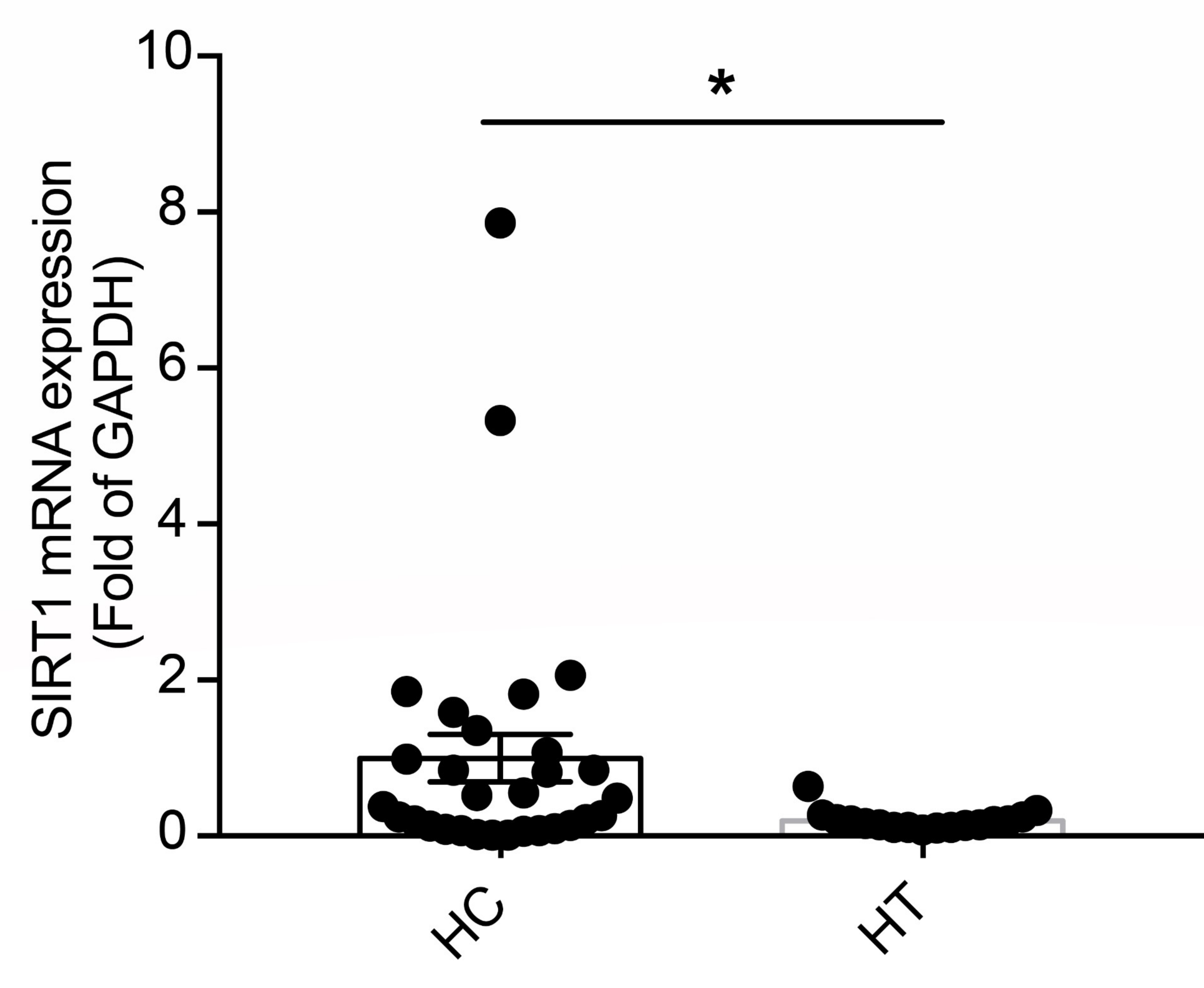

Supplement: Figure S2. The mRNA expression of SIRT1 was decreased in patients with Hashimoto thyroiditis. [file supplementary_figure_2.pdf]

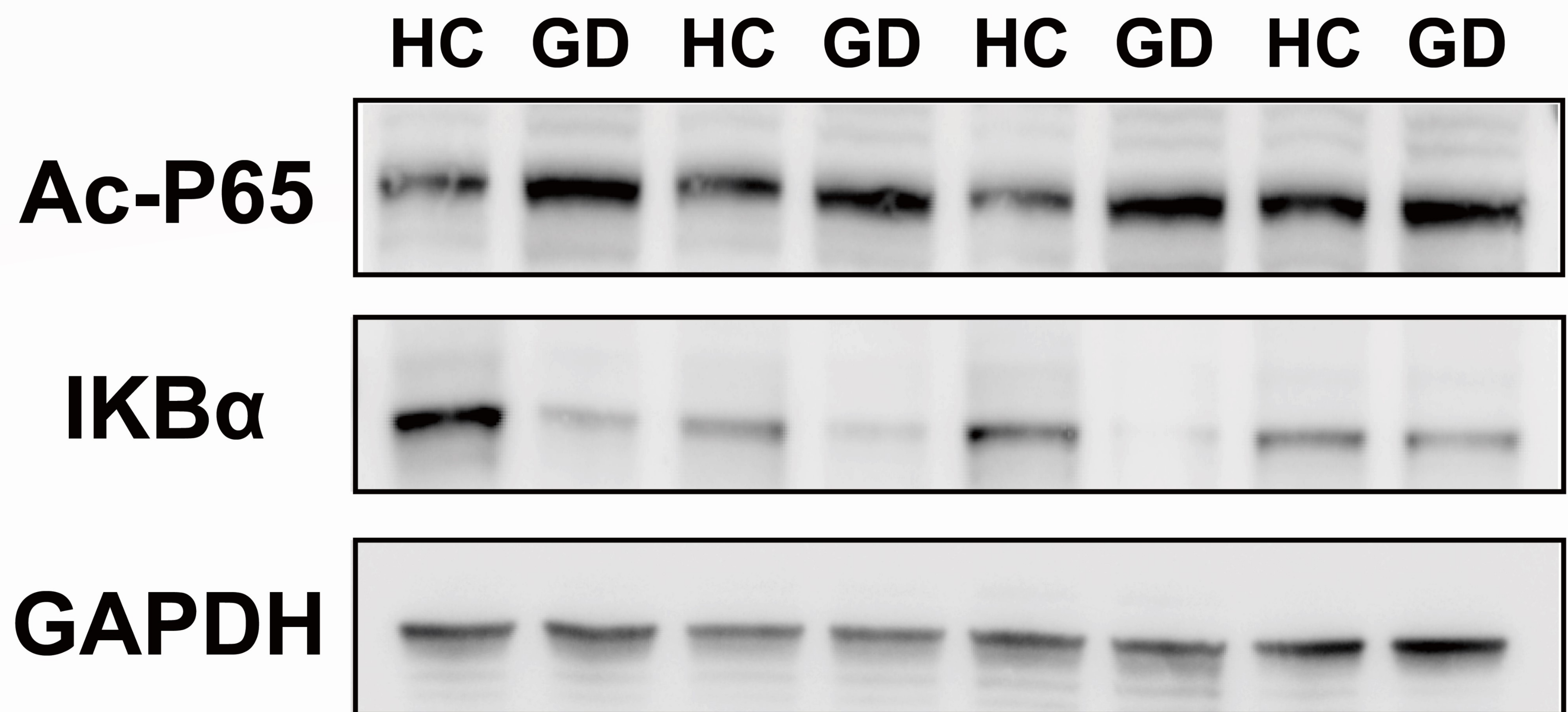

Supplement: Figure S3. Western blotting analysis of key molecules of the NF-κB pathway in GD patient and HC PBMCs. [file supplementary_figure_3.pdf]

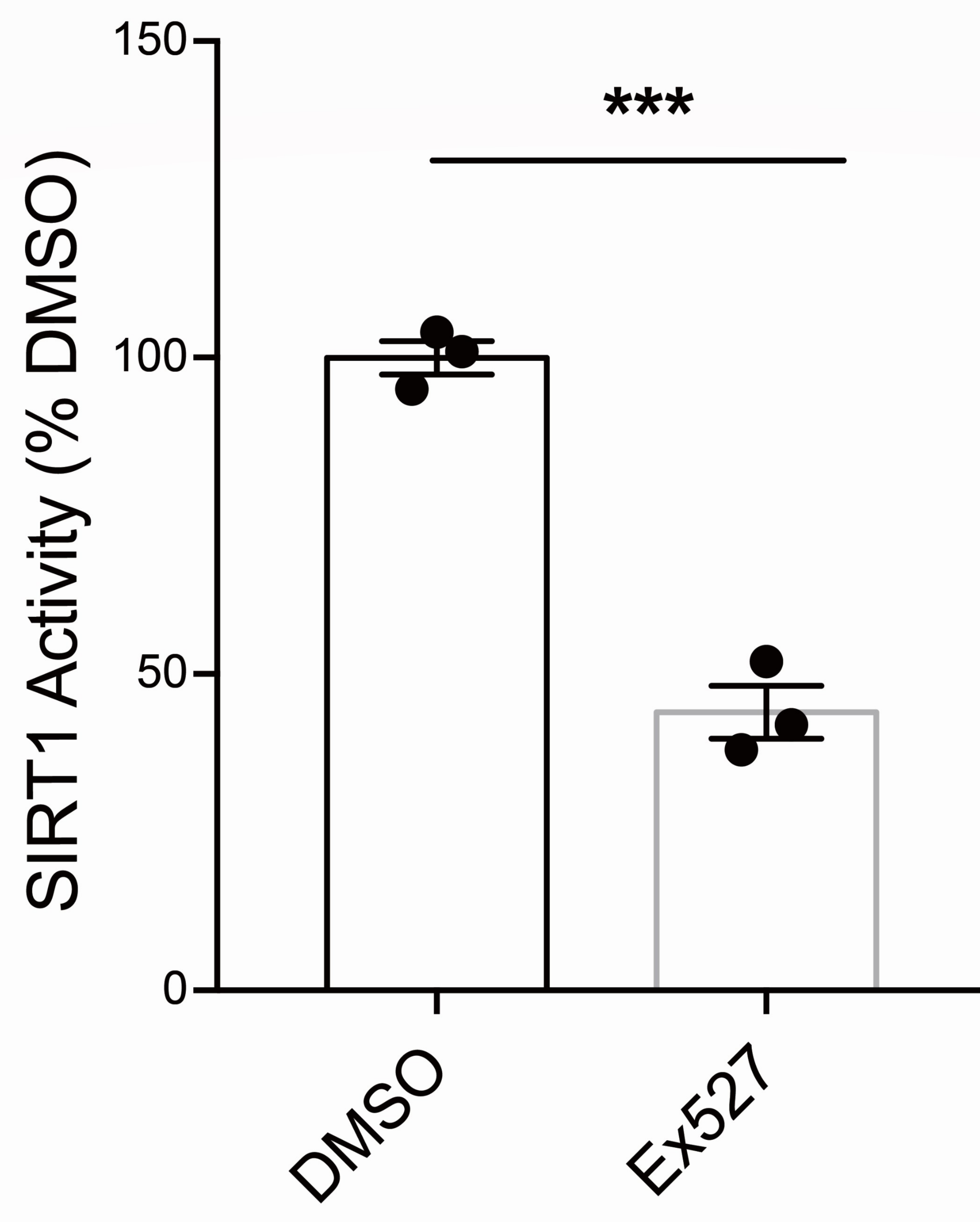

Supplement: Figure S4. SIRT1 activity was determined after treatment. [file supplementary_figure_4.pdf]

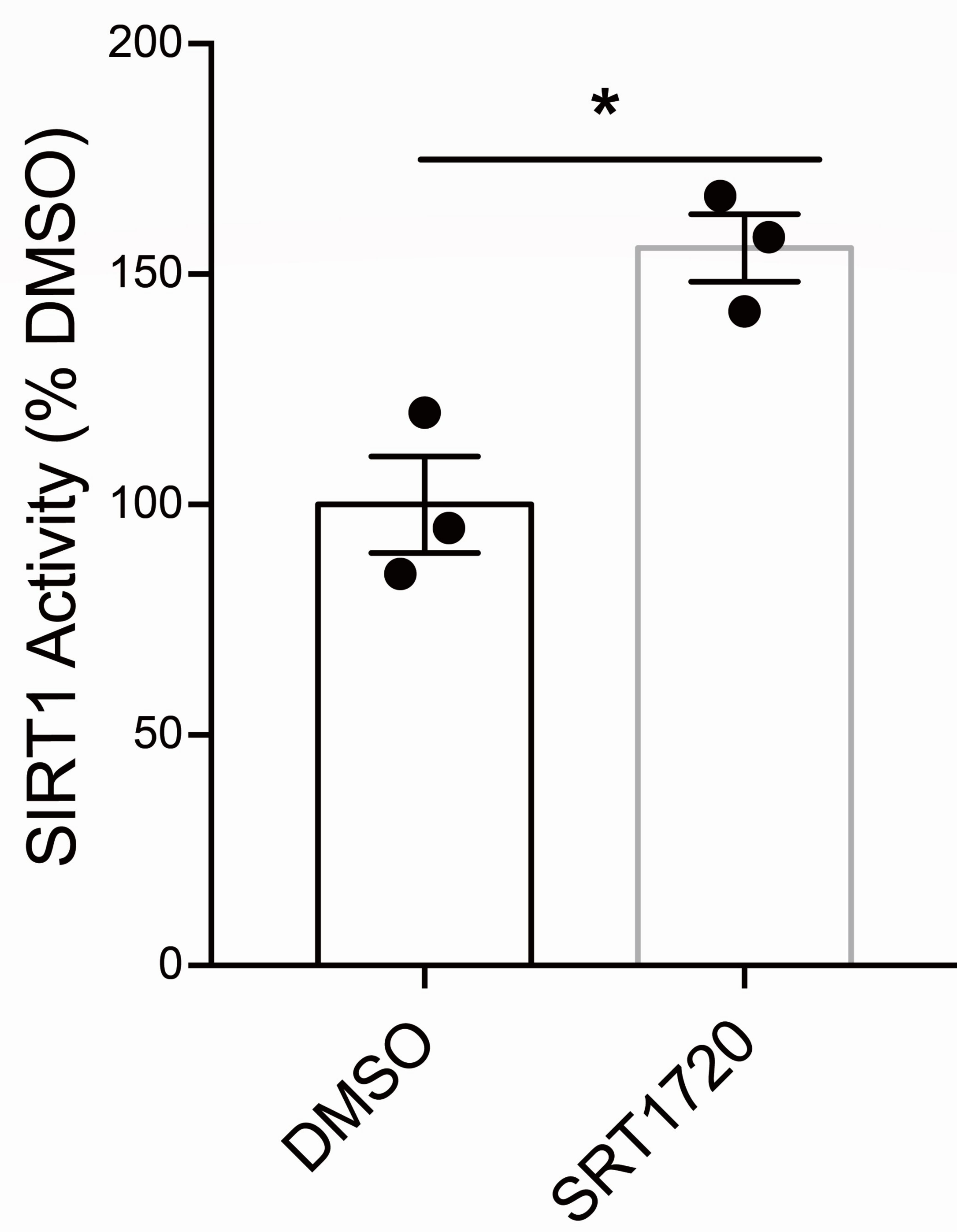

Supplement: Figure S5. Silencing effects of si-p65 were examined by qRT-PCR. [file supplementary_figure_5.pdf]
